# Metal-support interaction boosts the stability of Ni-based electrocatalysts for alkaline hydrogen oxidation

**DOI:** 10.1038/s41467-023-44320-w

**Published:** 2024-01-02

**Authors:** Xiaoyu Tian, Renjie Ren, Fengyuan Wei, Jiajing Pei, Zhongbin Zhuang, Lin Zhuang, Wenchao Sheng

**Affiliations:** 1 State Key Laboratory of Pollution Control and Resource Reuse, College of Environmental Science and Engineering, Tongji University, Shanghai Institute of Pollution Control and Ecological Security, Shanghai, 200092 PR China; 2 College of Chemistry and Molecular Sciences, Hubei Key Laboratory of Electrochemical Power Sources, Wuhan University, Wuhan, 430072 PR China; 3 Beijing Synchrotron Radiation Facility, Institute of High Energy Physics, Chinese Academy of Sciences, Beijing, 100049 PR China; 4 State Key Laboratory of Organic-Inorganic Composites, Beijing University of Chemical Technology, Beijing, 100029 PR China

**Keywords:** Electrocatalysis, Fuel cells

## Abstract

Ni-based hydrogen oxidation reaction (HOR) electrocatalysts are promising anode materials for the anion exchange membrane fuel cells (AEMFCs), but their application is hindered by their inherent instability for practical operations. Here, we report a TiO_2_ supported Ni_4_Mo (Ni_4_Mo/TiO_2_) catalyst that can effectively catalyze HOR in alkaline electrolyte with a mass activity of 10.1 ± 0.9 A g^−1^_Ni_ and remain active even up to 1.2 V. The Ni_4_Mo/TiO_2_ anode AEMFC delivers a peak power density of 520 mW cm^−2^ and durability at 400 mA cm^−2^ for nearly 100 h. The origin for the enhanced activity and stability is attributed to the down-shifted *d* band center, caused by the efficient charge transfer from TiO_2_ to Ni. The modulated electronic structure weakens the binding strength of oxygen species, rendering a high stability. The Ni_4_Mo/TiO_2_ has achieved greatly improved stability both in half cell and single AEMFC tests, and made a step forward for feasibility of efficient and durable AEMFCs.

## Abstract (web summary)

Nickel-based electrocatalysts for hydrogen oxidation in anion exchange membrane fuel cells face stability issues. Here the authors report Ni_4_Mo/TiO_2_ catalyst with significantly improved stability, owing to the efficient charge transfer from TiO_2_ to Ni.

## Introduction

Hydrogen-oxygen fuel cells with high energy efficiency and durability permit a sustainable energy system based on solar or electrical hydrogen converted from renewable energy resources^1^. With the fast development of low-cost oxygen reduction electrocatalysts at the cathode and alkane chain based anion exchange membranes^2,3^, enhancing the efficiency and durability of the anodic hydrogen oxidation reaction (HOR) electrocatalysts becomes particularly vital to the anion exchange membrane fuel cells (AEMFCs), as the HOR activity on the best Pt catalyst is two orders of magnitude slower in alkaline than in acidic medium^4,5^. Higher Pt catalyst loading would thus offset the cost merit brought by the cathode electrocatalysts and the membranes.

Enhancing the HOR activity of electrocatalysts is typically achieved by tuning the electronic structure of materials by alloying Pt with foreign metals. Pt alloys (PtRu, PtFe, PtCo, PtNi and PtCu) show greatly enhanced HOR activity compared to Pt, which is attributed to the optimized hydrogen binding energy (HBE)^6–8^ or potentially together with the optimized surface hydroxyl adsorption energy^9–11^. Similar strategy is also applied to developing non-precious HOR catalysts such as Ni to completely replace Pt group metals. In the few early attempts to improve the HOR activity of Ni, electrodeposited NiMo and CoNiMo thin films show superior activities to Ni, which is attributed to the optimized HBE introduced by the electronic effects between Ni and Mo/CoMo^12^. Recently, a group of Ni-based bimetallic nanoparticles, which are industry-relevant, have been developed for the HOR in base, showing much improved HOR activity with respect to Ni^13,14^. Except for tuning HBE on Ni, researchers also ascribe the improved HOR activity partially to the optimized surface hydroxyl group binding strength on foreign metals, which may facilitate the combination of -H and surface -‍OH group through the bi-functional mechanism^13^. Despite the debates in understanding the HOR mechanism in base, alloying achieves success in enhancing the overall HOR activities. However, non-precious metal catalysts often fail to meet the stability requirement. For example, the HOR performance on Ni-based catalysts usually dramatically drops at ~0.1 V versus the reversible hydrogen electrode (RHE) due to the Ni surface passivation^12,15^, while study shows that HOR catalysts should be able to remain stable up to at least 0.3 V for a substantial power density under practical operating conditions^16^. More severely, the anode potential would be driven up to ~0.8–0.9 V during the start-up and shut-down (SUSD) cycles or upon the H_2_ starvation events during fuel cell operations^17^. Recently, the amorphous Ni_52_Mo_13_Nb_35_ metallic glass was reported with the deactivation potential (the potential at which the HOR current starts to decrease) substantially increased to 0.8 V. Yet, the AEMFC using the Ni_52_Mo_13_Nb_35_ anode still shows a significant output cell voltage decay (~38%, from 0.74 to 0.46 V at 200 mA cm^−2^) in 50 h^18^. So far, none of the current Ni-based electrocatalysts can survive the harsh anodic conditions. Therefore, with achieved acceptable activity, efforts need to be geared to improve the stability of non-precious electrocatalysts.

The deactivation of HOR on Ni-based electrocatalysts is accompanied with Ni surface oxidation^19–21^, which passivates the catalyst surface and limits the electrochemically active window. Tuning the electronic structure of Ni-based materials for a weaker binding strength of Ni towards O/OH is therefore the most applied strategy. Ni_5.2_WCu_2.2_ ternary alloy^22^ and phase-separated Mo-Ni alloy (PS-MoNi)^23^ have exhibited elevated deactivation potential to ~0.3 V. Covering a protective layer outside Ni nanoparticles such as a few-layer hexagonal boron nitride (h-BN) could also prevent Ni from oxidation, which was speculated to originate from the weakened binding affinity towards oxygen species^24^. However, these negligible improvements in stability are not sufficient for practical AEMFC operation, and the stability issue is still the key problem to solve.

The interaction between the metal catalyst and the support also plays a vital role in tuning the reactivity of metal catalysts. Study shows that the interfacial charge transfer from Ni_3_N to the carbon support can move the deactivation potential from 0.16 V for Ni_3_N to 0.26 V for Ni_3_N/C^25^. Great improvements have been made on Pt group metals using metal oxide as the support. TiO_2_ partially encapsulated Pt shows extraordinary HOR activity above 1.0 V, wherein Pt alone will be oxidized and lose its HOR activity^26^. Using TiO_2_ to support Ru (Ru@TiO_2_) moves the deactivation potential from 0.2 V (Ru) to 0.9 V (Ru@TiO_2_)^27^. The interaction between Pt/Ru and TiO_2_, known as the metal-support interaction (MSI), enables an efficient charge transfer from TiO_2_ to metals as TiO_2_ is intrinsically an electron-rich semiconductor (negative semiconductor)^28^. Correspondingly, it is reasonable to speculate that the charge transfer introduced by the MSI may also exist between Ni-based non-precious metals and TiO_2_. Here, we report that the MSI between Ni_4_Mo and the TiO_2_ support boosts the durability for HOR up to 1.2 V, enabling a continuous AEMFC power output at 400 mA cm^−2^ for nearly 100 h, which makes a step forward for the automotive applications of the AEMFCs.

## Results

### HOR catalytic performance of Ni_4_Mo/TiO_2_

**Fig. 1:**
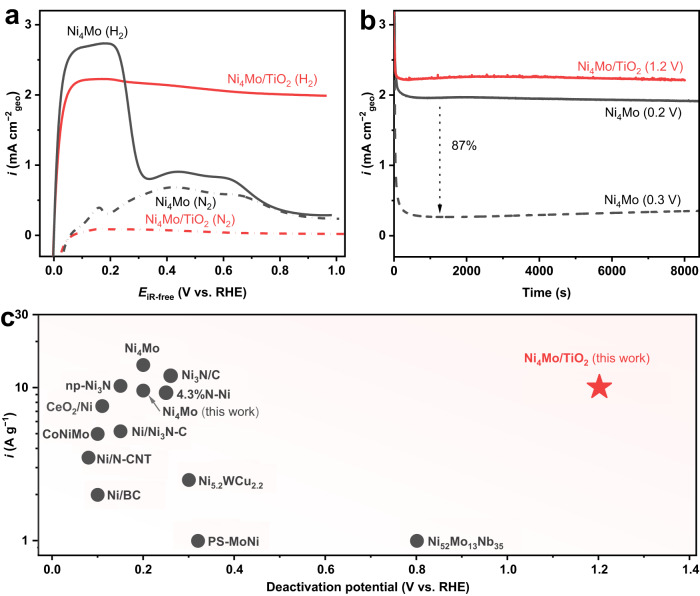
HOR performance of Ni_4_Mo/TiO_2_. **a** Positive-going sweeps of the cyclic voltammograms of Ni_4_Mo and Ni_4_Mo/TiO_2_ recorded in H_2_ and N_2_-‍saturated 0.1 M NaOH at 1600 r.p.m with a scanning rate of 0.5 mV s^−1^. The potentials are *iR*-corrected; **b** chronoamperometry curves of Ni_4_Mo and Ni_4_Mo/TiO_2_ at constant potentials in H_2_-saturated 0.1 M NaOH at 1600 ‍r.p.m. The potentials are not *iR*-corrected; and **c** deactivation potentials and mass activities of Ni-based non-precious metal electrocatalysts for the alkaline HOR. Details are listed in Supplementary Table S2. Note 1: The Ni loadings are 477 and 376 μg_Ni_ cm^−2^_geo_ for Ni_4_Mo and Ni_4_Mo/TiO_2_. Note 2: The data of CoNiMo^12^ and 4.3%N-Ni^79^ were calculated based on the original data. Note 3: The mass activities of bulky PS-MoNi^23^ and Ni_52_Mo_13_Nb_35_^18^ are unavailable. Here we set them at 1 A g^−1^ for comparison.

We prepared Ni_4_Mo/TiO_2_ catalysts through annealing the pre-mixed NiMo hydroxide precursor and the TiO_2_ support in H_2_ at 400 °C (see Methods). Optimization was made by adjusting the molar ratio between TiO_2_ and Ni_4_Mo according to their HOR performance (Supplementary Fig. S1 and Supplementary Table S1). The HOR catalytic performance of Ni_4_Mo and Ni_4_Mo/TiO_2_ catalysts was evaluated using the rotating disk electrode (RDE) method. Figure 1a shows the positive-going sweeps of the cyclic voltammograms (CVs) of Ni_4_Mo and the best performing Ni_4_Mo/TiO_2_ in both H_2_ and N_2_-saturated 0.1 M NaOH. Ni_4_Mo reaches the limiting current density of 2.65 mA cm^−2^_geo_ at only 85 mV overpotential, showing a very high HOR activity. Yet, the HOR current quickly drops at 0.2 V, and eventually tracks the CV curve collected in N_2_, which strongly suggests that the surface oxidation of Ni_4_Mo blocks the active surface areas and prohibits the hydrogen oxidation. In contrast, while Ni_4_Mo/TiO_2_ shows a similar HOR limiting current density of 2.23 mA cm^−2^_geo_ at 90 mV overpotential, it can catalyze the HOR even up to 1.0 V, with a mere 10% limiting current density decay. There is continuous HOR current at even ~1.4 V before oxygen starts to evolve (Supplementary Fig. S2). The difference in the polarization curves in H_2_ and N_2_-‍saturated electrolytes confirms that the anodic current in the presence of H_2_ in the full potential window is indeed originated from the H_2_ oxidation. It is also clearly seen from the CV curves collected in N_2_-saturated electrolyte that Ni_4_Mo/TiO_2_ hardly exhibits any features associated with the surface oxidation as Ni_4_Mo. Further chronoamperometry measurements verify that Ni_4_Mo/TiO_2_ exhibits a stable HOR current at as high as 1.2 V for 8000 s without noticeable decay (Fig. 1b and Supplementary Fig. S3). In comparison, Ni_4_Mo loses 87% of the HOR current when changing the holding potential from 0.2 to 0.3 V, and demonstrates an unacceptable low activity at 0.3 V (Fig. 1b).

Supplementary Fig. S4 shows the HOR polarization curves of Ni_4_Mo and Ni_4_Mo/TiO_2_ at different rotation speeds. Koutecky–Levich plots at 0.05 V exhibit a linear relationship between the inverses of *i* and *ω*^1/2^, with the slopes being 5.38 and 5.15 cm^2^ mA^−1^ s^−1/2^ for Ni_4_Mo and Ni_4_Mo/TiO_2_ (insets of Supplementary Fig. S4c, d). These values match reasonably well with the theoretical value of 4.87 cm^2^ mA^−1^ s^−1/2^ for the 2 e^−^ HOR^5^, and are also in close agreement with the previous study^29^. The exchange current density (*i*_0_) was extracted by fitting the kinetic current to the Butler–Volmer equation (Supplementary Fig. S5). The mass activity (*i*_0,m,298 K_) was then obtained by normalizing *i*_0_ to the Ni mass, as shown in Supplementary Table S1 and Supplementary Fig. S6. Ni_4_Mo has a *i*_0,m,298 K_ of 9.6 ± 0.5 A g^−1^_Ni_, in reasonably good agreement with previously reported values (6.8 A g^−1^_Ni_ in ref. ^13^ and 14.1 A g^−1^_Ni_ in ref. ^14^). The *i*_0,m,298 K_ of Ni_4_Mo/TiO_2_ first increases, and then decreases with increasing Ti/Ni ratio. At low Ti/Ni ratios (Ti/Ni < 0.4), Ni_4_Mo/TiO_2_ follows similar HOR behavior as Ni_4_Mo that it starts to deactivate at ~0.2 V (Supplementary Fig. S1), despite the slightly higher mass activities. The best performance of Ni_4_Mo/TiO_2_ is achieved at Ti/Ni = 0.42, wherein Ni_4_Mo/TiO_2_ exhibits a similar *i*_0,m,298 K_ (10.1 ± 0.9 A g^−1^_Ni_) as Ni_4_Mo, but a more stable HOR current above 1.0 V. When Ti/Ni ratio continues to increase (Ti/Ni > 0.5), in spite of its improved stability, Ni_4_Mo/TiO_2_ loses its mass activity significantly, which is most likely due to the lost active surface area by the TiO_2_ coverage. The HOR activation energies (*E*_a_) on Ni_4_Mo and Ni_4_Mo/TiO_2_, determined from the Arrhenius plots (Supplementary Fig. S7), are 15.9 kJ mol^−1^ and 19.5 kJ mol^−1^ respectively, matching well with 18.6 kJ mol^−1^ for Ni_4_Mo reported previously^13^. Both Ni_4_Mo and Ni_4_Mo/TiO_2_ demonstrate decreased *E*_a_ with respect to metallic Ni (30.0 kJ mol^−1^) and partially oxidized Ni (26.0 kJ mol^−1^)^30^.

Figure 1c and Supplementary Table S2 summarize recent important progress in the development of Ni-based non-precious metal electrocatalysts for the alkaline HOR. Despite previous efforts in improving the HOR activity by one order of magnitude, achievements in enhancing the deactivation potential remains very limited. With the acceptable high mass activity, the Ni_4_Mo/TiO_2_ reaches a new deactivation potential of 1.2 V for the alkaline HOR Fig. 1.

### AEMFC performance of the Ni_4_Mo/TiO_2_ anode catalyst

**Fig. 2:**
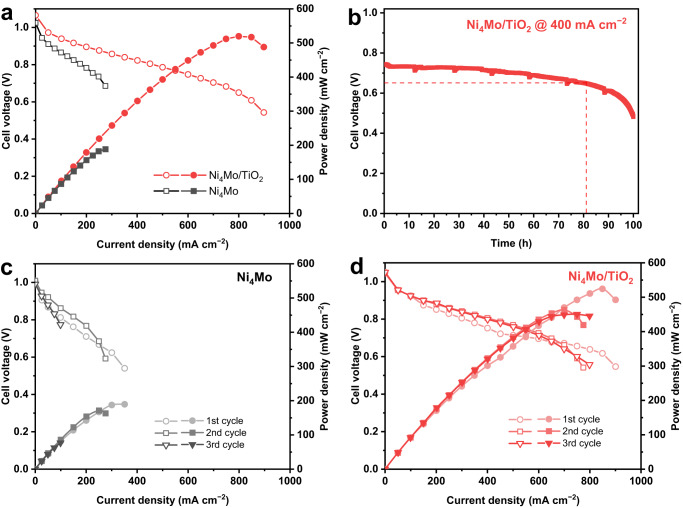
H_2_-O_2_ AEMFC performance. **a** Polarization and power density curves; **b** AEMFC durability of Ni_4_Mo/TiO_2_ at 400 mA cm^−2^; and **c**, **d** polarization and power density curves of Ni_4_Mo and Ni_4_Mo/TiO_2_ AEMFCs discharged for three times. AEMFC performance test conditions (**a**, **c** and **d**): cell temperature at 80 °C under H_2_ and O_2_ condition with a backpressure of 0.2 MPa for the anode and cathode, H_2_ and O_2_ humidified at 80 °C (100% RH) supplied to the anode and cathode compartments with a flow rate of 1000 sccm. AEMFC durability test conditions (**b**): H_2_ and O_2_ flow rate of 300 sccm and 500 sccm respectively, under otherwise identical conditions. The cell voltages were recorded with no *iR*-correction. The Ni loadings are 1.35 mg_Ni_ ‍cm^−2^ for Ni_4_Mo and Ni_4_Mo/TiO_2_ at the anode, and the Pt loading is 0.4 mg_Pt_ cm^−2^ for Pt/C at the cathode.

Encouraged by the excellent intrinsic HOR performance of the Ni_4_Mo/TiO_2_ catalyst in the RDE measurements, the assembled single cell test was conducted for AEMFC performance. The Ni_4_Mo and Ni_4_Mo/TiO_2_ were used as the anode catalysts, the commercial Pt/C was employed as the cathode catalyst, and QAPPT was applied as both anion exchange membrane and ionomer to fabricate the membrane electrode assembly (MEA). Figure 2a shows the cell voltage and power density of the cells using Ni_4_Mo and Ni_4_Mo/TiO_2_ as the anode catalysts. Ni_4_Mo approaches a peak power density (PPD) of 188 mW cm^−2^, which is prominent in Ni-based non-precious metals and is much higher than the NiMo/KB reported previously (120 mW cm^−2^)^15^. Strikingly, Ni_4_Mo/TiO_2_ boosts the PPD to 520 mW cm^−2^, among the best AEMFC performances that have been reported under similar conditions (see Supplementary Table S3 for a summary of AEMFC performance of non-precious metal anode catalysts). In addition, Ni_4_Mo can only approach a current density of 275 mA cm^−2^ (at 0.685 V), and fails to operate beyond this point because of the Ni oxidation induced deactivation. However, Ni_4_Mo/TiO_2_ is able to deliver a much higher current density of 900 mA cm^−2^ (at 0.543 V), indicating that Ni_4_Mo/TiO_2_ is more resistant to oxidation and remains active at a higher polarization potential. The long-term durability of Ni_4_Mo/TiO_2_ was evaluated at a large current density of 400 mA cm^−2^ (Fig. 2b), and the cell exhibits a stable operation for nearly 100 h. Gao et al. have demonstrated that the Ni@CN_x_ anode AEMFC exhibits a stable performance at 200 mA cm^−2^ for 100 h, which has been considered as a groundbreaking achievement at a large current density in AEMFCs^31^. The Ni_4_Mo/TiO_2_ anode further elevates the durability to a larger current density of 400 mA cm^−2^ for nearly 100 h (also see Supplementary Table S3 for Ni-based anode AEMFC durability). Moreover, the Ni_4_Mo/TiO_2_ anode can operate at 0.65 V (typical fuel cell operating potential for the automotive applications) for more than 80 h (Fig. 2b), paving the way for progress in the automotive applications of AEMFCs. Furthermore, the cells using Ni_4_Mo and Ni_4_Mo/TiO_2_ as the anode catalysts were discharged consecutively for three times. While the PPD of Ni_4_Mo is severely degraded from 188 mW cm^−2^ (1st cycle) to 171 mW cm^−2^ (2nd cycle) and 77 mW cm^−2^ (3rd cycle), as shown in Fig. 2c, Ni_4_Mo/TiO_2_ demonstrates a mere decrease of 7% in PPD from 520 mW cm^−2^ (1st cycle) to 480 mW cm^−2^ (2nd cycle), and retains the performance in the following cycle (Fig. 2d).

### Crystallographic and Morphologic Characteristics of Ni_4_Mo/TiO_2_

**Fig. 3:**
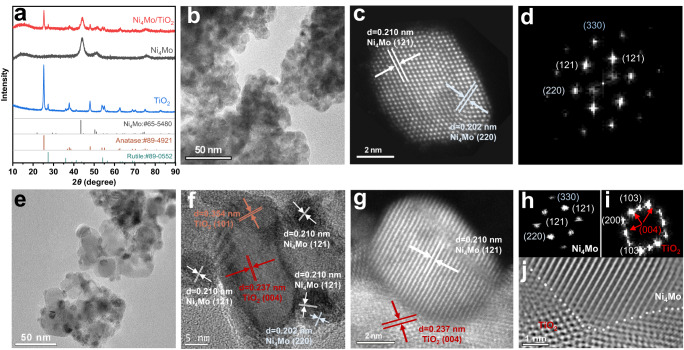
Physical characterizations. **a** XRD patterns of Ni_4_Mo, Ni_4_Mo/TiO_2_ and TiO_2_; **b** TEM image, **c** HAADF-STEM image, and **d** corresponding fast Fourier transform (FFT) pattern of Ni_4_Mo; **e** TEM image, **f** HR-TEM image, **g** HAADF-STEM image, **h** and **i** corresponding FFT patterns, and **j** inverse FFT pattern of Ni_4_Mo/TiO_2_.

The X-ray diffraction (XRD) pattern in Fig. 3a shows that the synthesized Ni and Mo alloy exhibits a Ni_4_Mo phase structure (JCPDS 65-5480), consistent with the ICP-OES results (Supplementary Table S1). The TiO_2_ distinctly presents both the anatase phase (JCPDS 89-4921) and the rutile phase (JCPDS 89-0552). The high intensity diffraction peaks at 25.3° and 37.8°, indexed to the anatase TiO_2_ (101) and (004) crystal planes, suggest that the anatase phase is the major composition in the pristine TiO_2_. Ni_4_Mo/TiO_2_ exhibits crystallographic features associated with both Ni_4_Mo and TiO_2_. Increasing the Ti/Ni ratio leads to the decreased relative peak intensity of Ni_4_Mo to TiO_2_ (Supplementary Fig. S8), corresponding to the gradually decreased Ni_4_Mo loading on TiO_2_. The transmission electron microscopy (TEM) image shows an interconnected particle morphology of Ni_4_Mo with the particle size of about 7.5 nm (Fig. 3b and Supplementary Fig. S9). Aberration-corrected high-angle annular dark-field scanning transmission electron microscope (HAADF-STEM) image of Ni_4_Mo (Fig. 3c) shows the 0.210 and 0.202 nm-lattice fringes, corresponding to the (121) and (220) planes of Ni_4_Mo. The corresponding fast Fourier transform (FFT) pattern demonstrate the tetragonal Ni_4_Mo crystalline phase with Ni_4_Mo (121), (220) and (330) planes (Fig. 3d). Elemental mappings using energy dispersive X-ray spectroscopy (EDS) further reveal the compositional distributions of Ni and Mo elements, confirming the formation of a uniform alloy (Supplementary Fig. S10). The pristine TiO_2_ has a rectangular plate morphology with the 0.354 and 0.237 nm-lattice fringes corresponding to the (101) and (004) planes of the anatase phase (Supplementary Fig. S11). TEM and HAADF-STEM images of Ni_4_Mo/TiO_2_ show a uniform distribution of spherical Ni_4_Mo particles with a mean size of 7.6 nm on the TiO_2_ support (Fig. 3e–g and Supplementary Fig. S12), maintaining the crystallographic characteristics as unsupported Ni_4_Mo with the lattice fringe interplanar spacings of 0.210 and 0.202 nm for Ni_4_Mo (121) and (220) planes. Besides, the lattice fringe with a spacing of 0.237 nm for the TiO_2_ support is consistent with the pristine TiO_2_, corresponding to the (004) plane. This structure was further identified by the FFT images (Fig. 3h, i). The inverse FFT image in Fig. 3j also show the interface between TiO_2_ and Ni_4_Mo, further demonstrating that Ni_4_Mo is supported on the TiO_2_.

### MSI in Ni_4_Mo/TiO_2_

Raman spectroscopy study of TiO_2_ shows six Raman-active modes at around 140 cm^−1^ (*E*_g_), 195 cm^−1^ (*E*_g_), 393 cm^−1^ (*B*_1g_), 511 cm^−1^ (*A*_1g_ + *B*_1g_) and 635 cm^−1^ (*E*_g_) (Supplementary Fig. S13a), in good agreement with Raman spectrum of the anatase TiO_2_^32^. No detectable Raman shift or peak broadening is observed for TiO_2_ treated at 400 °C in H_2_ (TiO_2_-H_2_-400 in Supplementary Fig. S13b), indicating that there are not enough oxygen vacancies being introduced, as they will cause the blue-shift and peak broadening in the Raman spectra of TiO_2_^33,34^. When Ni_4_Mo is decorated on the TiO_2_ support, there appears to be distinct blue-shift in the vibrational mode of *E*_g_ from 140 cm^−1^ (TiO_2_) to 150 cm^−1^ (Ni_4_Mo/TiO_2_) and peak broadening (Supplementary Fig. S13b), similar to that for Au or Ag decorated TiO_2_ in a previous study^35^. The blue-shift and peak broadening are most likely due to a compressive strain^36,37^, possibly introduced by lattice mismatch between the metal particles and the TiO_2_ support. The Raman results, together with the aforementioned electron microscopy study, confirm an interfacial interaction between the Ni_4_Mo particles and the TiO_2_ support.

**Fig. 4:**
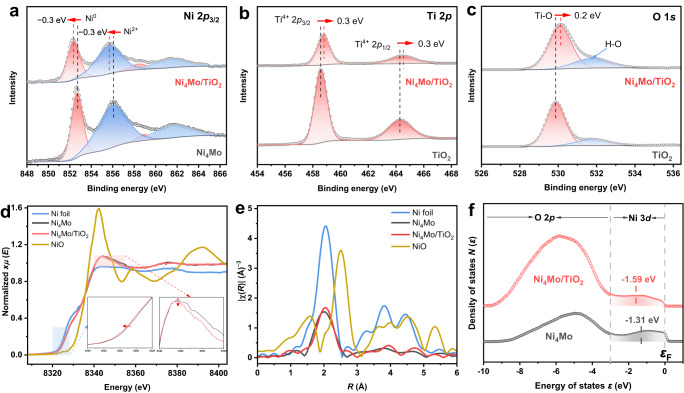
Metal-support interaction in Ni_4_Mo/TiO_2_. **a** Ni 2*p*_3/2_, **b** Ti 2*p* and **c** O 1*s* level X-ray photoemission spectra; **d** normalized Ni K-edge X-ray absorption spectra (the left inset is the magnified near-edge, and the right inset is the white line); **e** Fourier-transform of Ni K-edge EXAFS spectra; and **f** ultra-violet photoemission spectra of Ni_4_Mo and Ni_4_Mo/TiO_2_.

Figure 4a shows that X-ray photoemission spectrum (XPS) of the Ni 2*p*_3/2_ level of Ni_4_Mo contains four peaks, which can be assigned to Ni^0^ (852.7 eV), Ni^2+^ (856.1 eV) and their two satellites (858.9 eV for Ni^0^ satellite and 861.6 eV for Ni^2+^ satellite)^38–40^. Notably, both of the Ni^0^ and Ni^2+^ peaks of Ni_4_Mo/TiO_2_ at all Ti/Ni ratios shift negatively to lower binding energies (BE) (Fig. 4a and Supplementary Fig. S14a). XPS spectra of Mo 3*d* level reveal a complex Mo 3*d*_3/2_ and Mo 3*d*_5/2_ doublets, with the 3*d*_5/2_ peaks centered at 228.0, 229.0, 230.0 and 232.2 eV, corresponding to Mo^0^, Mo^4+^, Mo^5+^ and Mo^6+^ respectively (Supplementary Fig. S14b)^41–43^. These peaks do not shift when loading Ni_4_Mo onto TiO_2_ at all Ti/Ni ratios. Ti 2*p* doublets of TiO_2_ show two peaks centered at 458.6 and 464.3 eV, assigned to Ti^4+^ 2*p*_3/2_ and Ti^4+^ 2*p*_1/2_ respectively^44^, and the Ti^4+^ in Ni_4_Mo/TiO_2_ displays the positive core level shift to 458.9 eV (Ti^4+^ 2*p*_3/2_) and 464.6 eV (Ti^4+^ 2*p*_1/2_), indicating that Ti is in an electron-deficient status with a higher valence state (Fig. 4b). The O 1*s* spectrum for TiO_2_ is de-convoluted to two peaks at 529.9 and 531.2 eV (Fig. 4c), associated with the lattice oxygen in TiO_2_ (Ti-O) and the surface hydroxyl group (H-O)^27,44^. The O 1*s* spectrum of Ti-O for Ni_4_Mo/TiO_2_ also exhibits a positive BE shift by ~0.2 eV. Note that Ni_4_Mo/TiO_2_ experienced high temperature reduction in H_2_ at 400 °C; both of the Ti 2*p* and O 1*s* spectra of the bare TiO_2_ treated under the same condition (TiO_2_-H_2_-400) were also collected, and no significant difference was observed (Supplementary Fig. S15). At all Ti/Ni ratios, both of the Ti 2*p* and O 1*s* peaks shift to higher BE. The X-ray absorption fine structure spectroscopy (XAFS) is used to further probe the impact of the TiO_2_ support on the chemical environment of Ni. Notably, the absorption edge of Ni_4_Mo/TiO_2_ displays a slight shift toward the lower photon energy relative to Ni_4_Mo (Fig. 4d, left inset), indicating the electron enrichment on Ni atoms in Ni_4_Mo/TiO_2_. The white line absorption intensity is also weaker than that of Ni_4_Mo (Fig. 4d, right inset), signifying the lower Ni valence state in Ni_4_Mo/TiO_2_ (Supplementary Fig. S16). In addition, as shown in the Fourier-transform of Ni K-edge extended X-ray absorption fine structure (EXAFS) (Fig. 4e), the intensity of the peak at 2.0 Å, assigned to Ni-Ni/Ni-Mo coordination of Ni_4_Mo/TiO_2_, is higher than that of Ni_4_Mo, demonstrating an increased Ni coordination number, which is speculated to originate from the interaction with the TiO_2_ support. XPS and XAFS results strongly suggest that there exists electronic interaction between Ni_4_Mo and the TiO_2_ support through the charge transfer from TiO_2_ to Ni.

Work function values determined from ultra-violet photoemission spectroscopy (UPS) measurements also support the charge transfer from TiO_2_ (3.6 eV) to Ni_4_Mo (4.3 eV) (Supplementary Fig. S17). To collectively elucidate the electronic interaction between Ni_4_Mo and TiO_2_, the *d* band density of states was further investigated by UPS as shown in Fig. 4f and Supplementary Fig. S18. All samples illustrate intense emission between −3 and −10 eV below the Fermi level (*ε*_F_), related to O 2*p* orbitals^44,45^. TiO_2_ and TiO_2_-H_2_-400 do not show density of states at the *ε*_F_, but a very weak emission at −0.7 to −0.9 eV, which probably originates from the Ti^3+^ defect states^44^. The bands located at 0 to −3 eV are attributed to the Ni 3*d* states^46,47^. The Ni 3*d* band centers are roughly estimated to be −1.31 eV and −1.59 eV for Ni_4_Mo and Ni_4_Mo/TiO_2_ respectively, according to Eq. (1) (see Methods)^48–50^. Clearly, the centroid *d* band of Ni_4_Mo/TiO_2_ is regulated by TiO_2_, and down shifts away from the *ε*_F_ compared to Ni_4_Mo. Considering that the UPS measurement only probes the occupied *d* states, we later performed theoretical density functional theory (DFT) calculations to identify the density of states (DOS) including the unoccupied states in the next section.

### Robust structure of Ni_4_Mo/TiO_2_ in electrochemical measurements

Post-reaction characterizations were performed to study the structural stability of the Ni_4_Mo/TiO_2_ catalyst. After long-term stability test on the RDE, Ni_4_Mo/TiO_2_ still shows an interconnected particle morphology (TEM and HR-TEM in Supplementary Figs. S19a, b) with the Ni_4_Mo phase structure well maintained (selected-area electron diffraction, SAED in Supplementary Fig. S19c), and the Ni, Mo, Ti and O elements also have a uniform spatial distribution (EDS mappings in Supplementary Fig. S19d–h). XPS measurements similarly demonstrate inconspicuous change in the Ni 2*p*_3/2_, Mo 3*d*, Ti 2*p* and O 1*s* spectra after long-term stability test (Supplementary Fig. S20). ICP-MS results suggest that Ni and Ti dissolutions are negligible in 0.1 M NaOH. Yet, Mo is found to dissolve from the top surface of Ni_4_Mo particles (Supplementary Table S4), in good agreement with previously reported Mo dissolution on NiMo alloys^51–54^. The TiO_2_ support does not exhibit an inhibitive effect on Mo dissolution. However, Mo dissolution does not seem to deteriorate the HOR performance, as evidenced by the stable HOR current in the long term CA test (Fig. 1b).

After 100 h durability test in the AEMFC setup, the crystal, morphological, compositional, and electronic structures of Ni_4_Mo/TiO_2_ also exhibit negligible change (XRD, TEM, HR-TEM, SAED, EDS and XPS in Supplementary Figs. S21–23). The multiple post-reaction characterizations clearly demonstrate the structural robustness of Ni_4_Mo/TiO_2_, which enables its high HOR performance and durability in both RDE and AEMFC tests.

### Proposed mechanism for the enhanced stability of Ni_4_Mo/TiO_2_

The above results strongly suggest that the MSI between Ni_4_Mo and the TiO_2_ support modulate the electronic structure of Ni_4_Mo, causing a down-shifted *d* band center, which would weaken the adsorption strength of simple intermediate/molecule on Ni_4_Mo, such as H, O and CO, according to the *d* band theory^55^. Parallel hydrogen-temperature-programmed desorption (H_2_-TPD) results clearly demonstrate a lower desorption temperature on Ni_4_Mo/TiO_2_ than that on Ni_4_Mo (Supplementary Fig. S24), signifying a weakened hydrogen binding strength on Ni_4_Mo/TiO_2_. In line with the reduced hydrogen binding strength, although it is difficult to obtain the specific activity due to the uncertainty in the electrochemical surface area (ECSA) determination, it is reasonable to argue that Ni_4_Mo/TiO_2_ would exhibit a higher specific activity than Ni_4_Mo, as Ni_4_Mo/TiO_2_ has a similar Ni_4_Mo particle size and mass activity, but smaller ECSA due to the coverage of TiO_2_. Remarkably, oxygen-temperature-programmed oxidation (O_2_-TPO) results exhibit a much higher oxidation temperature of Ni_4_Mo/TiO_2_ than Ni_4_Mo (Supplementary Fig. S25), indicating a stronger oxidation resistance of Ni_4_Mo/TiO_2_, also in agreement with the hypothesis.

**Fig. 5:**
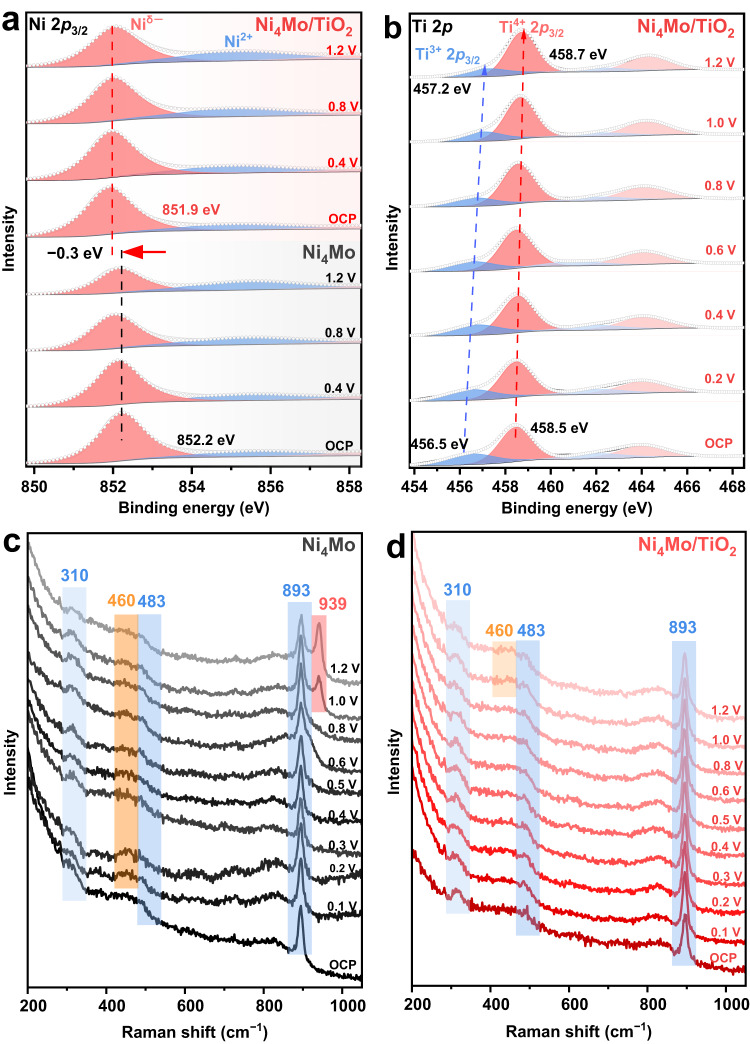
Mechanistic analyses. **a**, **b** Quasi in situ electrochemical XPS spectra, and **c**, **d** in situ electrochemical Raman spectra of Ni_4_Mo and Ni_4_Mo/TiO_2_ collected at the selected potentials in 0.1 M NaOH during the HOR. The potentials are *iR*-corrected.

Quasi in situ electrochemical XPS and in situ electrochemical Raman experiments were performed to explore the electronic structures and surface conditions of the Ni_4_Mo and Ni_4_Mo/TiO_2_ catalysts during the HOR process. After potential cycling between −0.1 to 0.2 V for a few cycles to active the surface, the Ni 2*p*_3/2_ and Ti 2*p* level XPS spectra were taken at the open circuit potential (OCP) and oxidizing potentials up to 1.2 V (Supplementary Figs. S26 and S27). The Ni 2*p*_3/2_ spectrum (Supplementary Fig. S26a) of Ni_4_Mo collected at OCP shifts by −0.5 eV relative to the ex situ experimental data as the Ni atoms are negatively charged during the surface activation process, and can be described as the electron-enriched Ni metal (Ni^δ−^)^56^. The BE of Ni^0^ (Ni^δ−^) 2*p*_3/2_ in both Ni_4_Mo and Ni_4_Mo/TiO_2_ stays constant at all potentials, while it is negatively shifted by 0.2-0.3 eV in Ni_4_Mo/TiO_2_ with respect to that in Ni_4_Mo regardless of the applied potentials (Fig. 5a and Supplementary Fig. S26a, b). The Ni^2+^ content estimated from XPS data is 21% in Ni_4_Mo at the initial OCP, and dramatically increases to 44% at 1.2 V, while the Ni^2+^ content is substantially reduced in Ni_4_Mo/TiO_2_ (21% at OCP to 33% at 1.2 V, Supplementary Fig. S26c). The correlation between the HOR polarization curves and the Ni^0^ (Ni^δ−^) percentages shows that the severely decreased current on Ni_4_Mo at 0.3 V matches relatively well with the decreased Ni^0^ (Ni^δ−^) content (Supplementary Fig. S26d), indicating that Ni_4_Mo deactivation is originated from Ni oxidation. The Ti^4+^ 2*p*_3/2_ spectra demonstrate a ~ 0.2 eV positive BE shift from OCP to 1.2 V (Fig. 5b and Supplementary Fig. S27a). Interestingly, a new peak signal in Ti 2*p* spectra of Ni_4_Mo/TiO_2_ emerges at the BE lower than Ti^4+^ 2*p*_3/2_ after the surface activation, which can be assigned to the reductive Ti^3+^ 2*p*_3/2_ species^44^. The Ti^3+^ 2*p*_3/2_ peak shifts to higher BE (456.5 eV at OCP to 457.2 eV at 1.2 V), and decreases in percentage (34% at OCP to 22% at 1.2 V, Supplementary Fig. S27b) with increasing applied potential. Ti^3+^ has been verified to boost the efficient charge transfer from TiO_2_ to metals by decreasing the work function of TiO_2_ bulk^57,58^, and thus may also play an important part in constructing the MSI of Ni_4_Mo/TiO_2_. The quasi in situ XPS experiments further confirm the existence of the charge transfer from TiO_2_ to Ni_4_Mo under the electrochemical conditions.

The in situ Raman spectra were also collected after the surface activation (Fig. 5c, d). The bands at 310 and 893 cm^−1^, which could be respectively assigned to the Mo = O stretching mode and bending mode in the MoO_4_^2−^ tetrahedron (see Supplementary Table S5 for the band assignment), exist in the full potential window investigated from the OCP to 1.2 V for both Ni_4_Mo and Ni_4_Mo/TiO_2_, owing to the oxidation of Mo in Ni_4_Mo to MoO_4_^2−^^52^. The peak at 483 cm^−1^ is indexed to the symmetric stretching mode of bridging Mo-O-Mo bond in Mo_2_O_7_^2−^, formed from the MoO_4_^2−^ dimerization^52,59^. Furthermore, the band at 460 cm^−1^ starting from 0.1 V and the one at 939 cm^−1^ starting from 1.0 V on Ni_4_Mo (Fig. 5c), attributed to the Ni-OH symmetric stretching mode of Ni(OH)_2_^60–63^ and Mo-O stretching mode of NiMoO_4_^59,64^ respectively, indicate the formation of hydroxide and oxide of Ni and Mo during the HOR from 0.1 to 1.2 V. Interestingly, these peaks associated with Ni-OH or Mo-O vibrations disappear on Ni_4_Mo/TiO_2_, and only when the potential reaches 1.0 V is the Ni-OH vibration marginally visible (Fig. 5d), revealing that most of the Ni surface atoms likely exist in the form of Ni^0^ (Ni^δ−^). These experimental results clearly suggest that Ni_4_Mo/TiO_2_ has great resistance to oxidation to endure the harsh oxidative conditions at high anodic potentials. This high oxidation resistance is likely due to the strong electronic modulation between Ni_4_Mo and TiO_2_, which renders a down-shifted *d* band center, and in turn weakened OH adsorption energy. DFT calculations also demonstrate a similar trend (Supplementary Fig. S28), in line with the hypothesis.

There have been debates as to the role of the surface hydroxyl species in the alkaline hydrogen oxidation on Pt group metal-based electrocatalysts, wherein the surface OH may (bi-functional mechanism^9^) or may not (HBE mechanism^4,65^) directly participate in the HOR process. However, on Ni-based non-precious materials, it is rather difficult to differentiate the role of the surface OH, as Ni-based materials usually exhibit complicated surface conditions in a broad potential regime, and there is by far no experimental evidence regarding the direct participation of surface OH in the HOR yet. In our opinion, the surface OH most likely acts as the spectator, which deteriorates the HOR activity by blocking the active surface area. This speculation is supported by the observations that the HOR current decrease on Ni_4_Mo and the metal surface oxidization occur concurrently (Fig. 1a and Supplementary Fig. S2), and the appearance of Raman bands associated with Ni(OH)_2_ and NiMoO_6_ in the Raman spectra of Ni_4_Mo. Therefore, it becomes very straightforward to weaken the OH binding strength in designing the HOR catalysts to enhance their anti-oxidation ability. By introducing the MSI using TiO_2_ as the support, the *d* band center of Ni_4_Mo is downwards shifted, causing a weakened binding strength to surface O or OH. It is clearly seen from Supplementary Fig. S29, the CV of Ni_4_Mo/TiO_2_ is mainly composed of the capacitive current, demonstrating much mitigated surface oxidation. Therefore, we hypothesize that the surface OH plays the role as the blocking species on Ni_4_Mo, and attribute the improved anti-oxidation ability of Ni_4_Mo/TiO_2_ to its much weakened OH adsorption strength.

### CO-tolerance of Ni_4_Mo/TiO_2_

The modulated *d* band of Ni_4_Mo by the TiO_2_ support not only weakens the OH binding strength, but may also reduce the CO binding energy^55^. As shown in Supplementary Fig. S30, the CO-stripping peak on Ni_4_Mo/TiO_2_ moves negatively to 0.50 V in comparison with 0.70 V on Ni_4_Mo. Moreover, Ni_4_Mo/TiO_2_ exhibits HOR activity with a negligible decay in the presence of 2000 p.p.m CO, compared to a ~14% decrease in the HOR limiting current density of Ni_4_Mo (Supplementary Fig. S31a). Chronoamperometry measurements at 0.2 V also demonstrate that the HOR current on Ni_4_Mo/TiO_2_ in the presence of 2000 p.p.m CO only decays 14% after 8000 s, much improved than a ~40% decay on Ni_4_Mo (Supplementary Fig. S31b). The satisfying CO-tolerant capability of Ni_4_Mo/TiO_2_ is attributed to its weakened CO binding strength, which originates from the modulated electronic structure of Ni_4_Mo by the TiO_2_ support.

### Applicability of the MSI to other Ni-based electrocatalyst

**Fig. 6:**
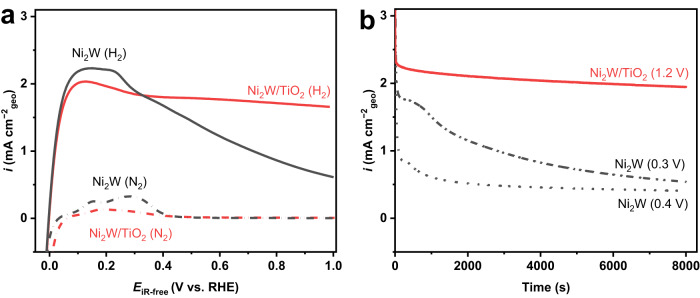
HOR performance of Ni_2_W/TiO_2_. **a** Positive-going sweeps of the cyclic voltammograms of Ni_2_W and Ni_2_W/TiO_2_ recorded in H_2_ and N_2_-‍saturated 0.1 M NaOH at 1600 r.p.m with a scanning rate of 0.5 mV s^−1^. The potentials are *iR*-corrected; and **b** chronoamperometry curves of Ni_2_W and Ni_2_W/TiO_2_ in H_2_-saturated 0.1 M NaOH at 1600 r.p.m. The potentials are not *iR*-corrected. The Ni loadings are 349 and 312 μg_Ni_ cm^−2^_geo_ for Ni_2_W and Ni_2_W/TiO_2_.

We further investigated whether the MSI between the metal catalyst and the TiO_2_ support can be applied to other Ni-based electrocatalysts for the enhanced stability. Despite its excellent HOR activity, Ni_2_W also loses the HOR activity at potentials higher than 0.2 V, in line with the surface oxidation in this potential regime (Fig. 6a). Optimized Ni_2_W/TiO_2_ (Ti/Ni = 0.46) not only exhibits good HOR activity at a low overpotential, but maintains the activity at a very small degradation rate (Fig. 6a and Supplementary Fig. S32, also see Methods, Supplementary Table S6, Supplementary Figs. S33–36 for detailed information on the synthesis and material structure). Although W is also found to slightly leach from the surface of Ni_2_W nanoparticles in the alkaline electrolyte under the experimental conditions (Supplementary Table S7), long-term stability tests confirm that Ni_2_W/TiO_2_ can remain stable activity towards hydrogen oxidation at 1.2 V (Fig. 6b and Supplementary Fig. S37). This finding highlights the strategic importance of the MSI in tailoring the electronic structure of Ni-based electrocatalysts to elevate the HOR stability in alkaline electrolytes.

In conclusion, by applying the MSI between NiM alloys and the TiO_2_ support, we successfully synthesized Ni_4_Mo/TiO_2_ and Ni_2_W/TiO_2_ electrocatalysts with enhanced HOR stability. The catalysts not only maintain good HOR mass activities (10.1 ± 0.9 A g^−1^_Ni_ for Ni_4_Mo/TiO_2_ and 6.8 ± 0.2 A g^−1^_Ni_ for Ni_2_W/TiO_2_), but also show stable HOR current at as high as 1.2 V. AEMFC tests, using Ni_4_Mo/TiO_2_ as the anode catalyst, demonstrate a good performance with a peak power density of 520 mW cm^−2^ and durability at 400 mA cm^−2^ for nearly 100 h. Detailed structural and electronic analyses confirm the existence of the MSI through charge transfer from the TiO_2_ support to NiM metals. Modulated electronic structure of NiM weakens the adsorption strength of H, O/OH and CO on NiM surfaces, rendering a high anti-oxidation ability of NiM/TiO_2_. These results highlight the significance of the MSI in improving the catalyst stability, and evidence the intriguing promise of Ni_4_Mo/TiO_2_ as an efficient and robust anode catalyst, making a step forward in the applications of the AEMFC technology.

## Methods

### Material synthesis

The Ni_4_Mo/TiO_2_ catalysts were synthesized through a three-step method. Firstly, the NiMo hydroxide precursor was prepared by a solvothermal method^14^. In brief, 735 mg Ni(NO_3_)_2_·6H_2_O (98%, Sinopharm) and 81 mg (NH_4_)_6_Mo_7_O_24_·4H_2_O (99%, Alfa Aesar) were dissolved in 2.5 mL ultra-pure H_2_O (18.2 MΩ cm, Millipore), followed by addition of 12.5 mL ethylene glycol (99%, Innochem) and 1 mL NH_3_·H_2_O (18% NH_3_ basis, Sigma-Aldrich). After stirring for 2 h, the solution was transferred into a Teflon-lined stainless steel autoclave and heated at 190 °C for 1 h. When cooling down to the room temperature, the synthesized green powder was washed using ethyl alcohol (99.5%, Innochem) and ultra-pure H_2_O (1:1 in volume) for five times, and collected by centrifugation. Secondly, six batches of the green precipitate were mixed with 100, 67, 50, 33, 25 and 20 mg TiO_2_ (Aeroxide® P25, Acros Organic) respectively, and then dispersed in 50 mL ethyl alcohol by magnetically stirring for 20 h. Subsequently, the mixture was collected by vacuum filtration, and grounded in a mortar for 20 min after drying at 50 °C in vacuum (2.13 × 10^4^ Pa). Finally, the collected substance was heated up to 400 °C at 3 °C min^−1^ in a reductive atmosphere (H_2_/N_2_ = 1:5 in volume) and remained at 400 °C for 1 h to obtain the Ni_4_Mo/TiO_2_ catalysts. Ni_4_Mo was prepared using the same method without adding TiO_2_ in the second step.

Ni_2_W and Ni_2_W/TiO_2_ catalysts were synthesized by the same procedure, except that the initial Ni and W precursors were 735 mg Ni(NO_3_)_2_·6H_2_O (98%, Sinopharm) and 177.5 mg (NH_4_)_2_WO_4_ (99.99%, Alfa Aesar), and the final heat-treatment temperature was 500 °C.

### Physical characterizations

#### Electron microscopy

High-resolution transmission electron microscopy (HR-TEM) measurement was conducted on the FEI Talos 200X and JEOL JEM-2100F transmission electron microscopes at the accelerating voltage of 200 kV. Aberration-corrected high-angle annular dark-field scanning transmission electron microscopy (HAADF-STEM) measurement was carried out on an atomic-resolution analytical microscope (thermalfisher scientific titan themsis Z) equipped with a probe spherical aberration corrector at an acceleration voltage of 300 kV. Energy dispersive X-ray spectroscopy was collected on the Oxford Instruments X-Max 80 T. The sample was made through dispersing the catalysts in ethyl alcohol by sonication. The dispersion was then dropped on an ultrathin carbon grid, and dried in air for few minutes.

#### Powder X-ray diffraction (XRD)

XRD measurement was performed on a D8 Advance X-ray diffractometer (Bruker) using Cu Kα radiation (*λ* = 0.15418 nm) at 40 kV and 40 mA. The data were collected with 2*θ* ranged from 10° to 90° at a scanning rate of 10° min^−1^.

#### Raman spectroscopy

Raman experiment was carried out with a confocal microscope Raman spectrometer (DXR3, Thermo Scientific). The excitation wavelength was 532 nm.

#### X-ray photoemission spectroscopy (XPS)

The states of Ni, Mo, Ti and O in the catalysts were examined on a ThermoFischer ESCALAB 250Xi X-ray photoelectron spectrometer equipped with Al X-ray source (Al Kα, 1486.6 eV). All spectra were processed using the Shirley background correction, and calibrated with the C 1*s* component at 284.8 eV. The Gaussian–Lorentzian line shape was adopted to fit the spectra.

#### X-ray absorption fine structure spectroscopy (XAFS)

The XAFS measurements were performed at 1W1B station in Beijing Synchrotron Radiation Facility, operated at 2.5 GeV with a maximum current of 250 mA. The spectra were collected in the fluorescence mode using a Lytle detector. Samples were pelletized with diameter of 8 mm and thickness of 1 mm using the PVDF powder as the binder. The acquired Ni K-edge extended X-ray absorption fine structure (EXAFS) data were processed according to the standard procedures using the Athena and Artemis implemented in the IFEFFIT software packages^66^. The EXAFS spectra were subtracted by the post-edge background from the overall absorption, and normalized with respect to the edge-jump step. The χ(*k*) data were Fourier transformed to real (*R*) space using a hanning window (d*k* = 1.0 Å^−1^) to separate the EXAFS contributions from different coordination shells. Least-squares curve fitting was performed using the Artemis module of IFEFFIT software packages to obtain the quantitative structural parameters. In the fitting, the amplitude reduction factor *S*_0_^2^ was fixed, and the internal atomic distances *R*, Debye-Waller factor *σ*^2^, and the edge-energy shift Δ*E*_0_ were allowed to run freely.

#### Ultraviolet photoemission spectroscopy (UPS)

Surface electronic structure of the catalysts was investigated on the PHI5000 VersaProbe III electron spectrometer (Scanning ESCA Microprobe) at UV photon energy of 21.2 eV (He I) under ultra-high vacuum (4 ×10^−6^ Pa). The total energy resolution was 0.10 eV. Shirley background was subtracted as described in the previous study^48^. The band between −3 eV and −10 eV centered at −4.9 eV for the Ni_4_Mo sample was assigned to the O 2*p* states due to inevitable oxidation upon exposure to air^45^, while the band in the same window centered at −6.0 eV for the Ni_4_Mo/TiO_2_ sample was O 2*p* states mainly originated from TiO_2_^44^. The bands ranged from 0 to −3 eV in both samples were assigned to the Ni 3*d* photoemission^46^. The *d* band center energy relative to the Fermi level was calculated based on the following equation^48–50^

1 $${\varepsilon }_{{{\rm{d}}}}=\int N(\varepsilon )\varepsilon {{{\rm{d}}}} \varepsilon \Big/\int N(\varepsilon ) {{{\rm{d}}}}\varepsilon$$

where *N(ε)* is the density of states, and *ε* is the energy of states.

For the work function calculation, the valence band spectra were also collected at the same spectrometer with a − 5 V bias voltage applied to the sample. The work function ($\varphi$) could be calculated from the following equation^67^

2 $$\varphi=h\nu -{E}_{{{\rm{cutoff}}}}$$

where *hν* is the energy of the UV photon (He I, 21.2 eV), and *E*_cutoff_ is the energy of the secondary-electron cutoff.

#### H_2_-temperature programmed desorption (H_2_-TPD)

Surface property towards H adsorption/desorption of the catalyst was measured using a ChemBET instrument (Quantachrome). The signal was determined by a thermal conductivity detector (TCD). 100 mg catalyst was placed in a quartz tube, and heated from the room temperature up to 400 °C at a ramping rate of 10 °C min^−1^ with flowing He. The sample was kept at 400 °C for 30 min, and then cooled down to the room temperature under He-flow. Subsequently, H_2_ was introduced into the tube for adsorption until a stable TCD signal. After removing H_2_ with flowing He, the sample was heated up to 400 °C with a ramping rate of 10 °C min^−1^.

#### O_2_-temperature programmed oxidation (O_2_-TPO)

Surface property towards binding strength of oxygen-related species was measured on the same instrument as H_2_-TPD. 100 mg catalyst was placed in a quartz tube, and heated from the room temperature up to 400 °C at a ramping rate of 10 °C min^−1^ with flowing He. The sample was kept at 400 °C for 30 min, and then cooled down to the room temperature under He-flow. Subsequently, the sample was gradually oxidized from the room temperature to 400 °C at a ramping rate of 10 °C min^−1^ in the presence of 3% O_2_ in He.

#### Inductively coupled plasma-optical emission spectrometry (ICP-OES)

The chemical contents of Ni, Mo and Ti were examined by ICP-OES on Agilent-730-OES. In brief, the catalyst was mixed with 5 mL HNO_3_ (67%, Sinopharm), 1 mL HF (40%, Sinopharm) and 1 mL HCl (37%, Sinopharm) in a Teflon-lined stainless steel autoclave, followed by heating at 180 °C for 8 h. After cooling down to the room temperature, the solution was transferred to a 25-mL volumetric flask, and diluted with ultra-pure H_2_O to the metered volume for ICP measurement.

#### Inductively coupled plasma mass spectrometry (ICP-MS)

ICP-MS (Aglient-7700) was used to detect the Ni, Mo, W and Ti dissolution in the alkaline electrolyte after electrochemical measurements.

### Electrochemical measurement

All electrochemical measurements were performed on the electrochemical workstation (VSP-300, Biologic). The temperature was set at 25 ± 0.5 °C, unless otherwise emphasized. The catalyst ink was prepared by dispersing and sonicating the catalyst in a mixture of 750 μL isopropanol (99.5%, Innochem), 200 μL ultra-pure H_2_O and 50 μL Nafion (5 wt%, Sigma-Aldrich) with a final concentration of 6-8 mg_Ni_ mL^−1^. 10 μL catalyst ink was deposited on a glassy carbon electrode (5 mm in diameter, Tianjin Aida), which was pre-polished with 50 nm alumina slurry (99.0%, Tianjin Aida), and dried in air at the room temperature, resulting in the Ni loading of ~400 μg_Ni_ cm^−2^_geo_ (see Supplementary Tables S1 and S6 for the specific loadings). The catalyst thin film electrode was then mounted onto a rotator (Pine Instrument), serving as the working electrode. A KCl-saturated calomel electrode (SCE, Tianjin Aida) and a graphite rod (spectral purity, Tianjin Aida) were used as the reference and counter electrodes, respectively. All potentials reported in this paper were referenced to the reversible hydrogen electrode (RHE), which was calibrated by measuring the HER/HOR on a Pt disk (5 mm in diameter, Pine Instrument) in H_2_-saturated 0.1 M NaOH (99.99% metal trace, Sigma-Aldrich).

#### Cyclic voltammetry measurement

Surface properties of the as-prepared electrocatalysts were investigated using the cyclic voltammetry (CV) method from −0.3 V to 1.8 V versus RHE with a rotating speed of 1600 r.p.m and a scanning rate of 20 mV s^−1^ in N_2_-saturated 0.1 M NaOH.

#### Rotating disk electrode measurement

HOR performances of the as-prepared electrocatalysts were examined using the RDE technique. The catalyst thin film electrodes were first activated by CV in H_2_-saturated 0.1 M NaOH between −0.1 and 0.2 V versus RHE at 20 mV s^−1^ for several cycles until a steady polarization curve was obtained. The HOR polarization curves were then collected with a rotating speed of 400, 900, 1600 and 2500 r.p.m and a scanning rate of 0.5 mV s^−1^ to minimize the capacitive charge contribution.

The HOR kinetic current (*i*_K_) was calculated based on the Koutecky–Levich equation^68^

3 $$\frac{1}{i}=\frac{1}{{i}_{{{{{\rm{K}}}}}}}+\frac{1}{{i}_{{{{{\rm{D}}}}}}}$$

where *i* is the measured current, and *i*_D_ is the diffusion limited current.

The HOR exchange current (*i*_0_) was obtained subsequently by fitting *i*_K_ to the Butler–Volmer equation^69^

4 $${{i}}_{{{{{\rm{K}}}}}}={{i}}_{0}\left({{e}}^{\frac{{{{{{{\rm{\alpha }}}}}}}_{{{{a}}}}{F}}{{RT}}{\eta }}-{{e}}^{\frac{-(1-{{{{{{\rm{\alpha }}}}}}}_{{{{a}}}}){F}}{{RT}}{\eta }}\right)$$

where *i*_0_ is the HOR exchange current, *α*_a_ is transfer coefficient for the HOR, *F* is the Faraday constant (96485 C mol^−1^), *R* is the universal gas constant (8.314 J mol^−1^ K^−1^), *T* is the temperature, and *η* is the overpotential. *α*_a_ was between ~0.1 and ~0.4.

Activation energy (*E*_a_) of the HOR was measured at different temperatures from 2 °C to 30 °C. The *E*_a_ can be calculated from the Arrhenius equation

5 $$\log {i}_{0}=\frac{-{E}_{{{{a}}}}}{{{{{\mathrm{ln}}}}}10\times R\times T}{{{{\rm{+const.}}}}}$$

where *i*_0_ (mA cm^−2^) is the exchange current density at different temperatures, *E*_a_ is the activation energy (J mol^−1^), *R* is the universal gas constant (8.314 J mol^−1^ K^−1^), and *T* is the temperature (K).

#### Chronoamperometry measurement

Chronoamperometry (CA) experiment was taken at a constant potential for 8000 s in H_2_/N_2_-saturated 0.1 M NaOH with a rotating speed of 1600 r.p.m for stability test.

#### CO stripping and CO tolerance measurement

CO stripping was conducted using the CA and CV methods. The potential was held at 0.1 V versus RHE for 30 min, during which time the electrolyte was purged with CO, allowing for complete CO adsorption, followed by 20 min-purging with N_2_ to remove the remaining CO in the electrolyte. Then CO stripping was performed by taking CV from 0 to 1.2 V versus RHE at a scanning rate of 20 mV s^−1^ for 2 cycles. CO tolerance experiment was performed using the RDE technique and CA method in H_2_-saturated 0.1 M NaOH at 0.2 V versus RHE in the presence of 2000 p.p.m CO.

#### Electrochemical impedance spectroscopy (EIS) measurement

Solution resistance was obtained after each RDE test by EIS measurement. The EIS spectra were taken at 0 V vs. open circuit potential (OCP) with a 10 mV voltage perturbation, and the frequency was from 100 mHz to 200 kHz. The real part of the resistance at 1 kHz was taken as the solution resistance (*R* ≈ 40 Ω).

### In situ electrochemical Raman spectroscopy test

The in situ electrochemical Raman test was performed on a Renishaw Via Raman microscope with a 532 nm excitation wavelength and a 50× objective. The sample ink was prepared by dispersing the catalyst in a mixture of ultra-pure H_2_O, isopropanol and Nafion, and then dropped on a piece of carbon paper (CP, 15 × 15 mm^2^), serving as the working electrode. Prior to collecting the Raman data, the Ni_4_Mo and Ni_4_Mo/TiO_2_ working electrodes were scanned between −0.1 and 0.2 V versus RHE for 10 cycles in H_2_-saturated 0.1 M NaOH in a home-made Raman cell (Supplementary Fig. S38), equipped with a platinum counter electrode and a SCE reference electrode. The solution resistance was ~26 Ω for the in situ Raman setup. The working electrode was held at each potential (OCP and 0–1.5 V versus RHE at an interval of 0.1 V) for 2 min for the Raman spectra collection. The laser intensity was set to be 10% with a collection time of 50 s for each spectrum.

### Quasi in situ electrochemical X-ray Photoemission Spectroscopy test

Quasi in situ electrochemical XPS measurement was carried out on the ThermoFischer ESCALAB 250Xi instrument by applying a monochromatic Al Kα X-ray source (1486.8 eV) operating at 12.5 kV and 16 mA under ultra-high vacuum (8 × 10^−10^ Pa). The total energy resolution was 0.10 eV. The catalyst ink was prepared by dispersing the catalyst in a mixture of ultra-pure H_2_O, ethyl alcohol and Nafion, and then dropped on a glassy carbon (GC) electrode (4 mm in diameter), serving as the working electrode. Before collecting the XPS data, the Ni_4_Mo and Ni_4_Mo/TiO_2_ working electrodes were scanned between −0.1 and 0.2 V versus RHE for 10 cycles in H_2_/N_2_ mixture-saturated 0.1 M NaOH in a home-made cell (Supplementary Fig. S39), equipped with a platinum counter electrode and a SCE reference electrode. The solution resistance was ~10 Ω for the quasi in situ XPS setup. The working electrode was first held at each potential (OCP, 0, 0.2, 0.3, 0.4, 0.5, 0.6, 0.8, 1.0 and 1.2 V versus RHE) for 2 min, then vacuumed in the preparation chamber, and finally transferred to the test chamber for XPS spectrum collection without exposure to the air. All spectra were processed using the Shirley background correction, and calibrated with the C 1*s* component at 284.8 eV. The Gaussian–Lorentzian line shape was adopted to fit the spectra.

### Membrane-electrode assembly and AEMFCs test

The as-synthesized Ni_4_Mo or Ni_4_Mo/TiO_2_ was used as the anode catalyst and the commercial Pt/C (60 wt%, Johnson-Matthey) was used as the cathode catalyst. The self-designed QAPPT [quaternary ammonia poly (N-methyl-piperidine-co-p-terphenyl)] was applied as both anion exchange membrane and ionomer in the electrodes^70^. For the anode catalyst, the ink was prepared by adding Ni_4_Mo or Ni_4_Mo/TiO_2_, Vulcan XC-72 carbon and 20 mg mL^−1^ ionomer (16 wt%) solution into isopropanol and then sonicated for 30 min. The weight ratio of Ni_4_Mo or Ni_4_Mo/TiO_2_ to Vulcan XC-72 carbon was 4:1. For the cathode catalyst, Pt/C and the 20 wt% ionomer solution was mixed in isopropanol and then sonicated for 15 min. To make the catalyst-coated membrane (CCM), the catalyst ink was sprayed onto the membrane (25 μm), which was heated at 70 °C to remove the isopropanol. The anode and cathode catalyst loadings were 1.35 mg_Ni_ cm^−2^ and 0.4 mg_Pt_ cm^−2^ respectively, and the catalyst sprayed area was fixed to 4 cm^2^. In order to exchange the anion of the membrane and ionomer to OH^−^, the CCM was soaked in 1 M KOH at 60 °C for 24 h, and meanwhile hydrogen was purged into the KOH solution to prevent the catalyst oxidation. Subsequently, the CCM was washed with ultra-pure H_2_O for several times to remove the excess KOH. The membrane electrode assembly (MEA) was assembled by placing the CCM between two pieces of carbon paper (AvCarb GDS3250) used as gas diffusion layer without hot-pressing. The AEMFC performance test was conducted on an 850E Multi Range fuel cell test station (Scribner Associates, USA). The test was operated at 80 °C under H_2_ and O_2_ condition with a backpressure of 0.2 MPa on the anode and cathode. H_2_ and O_2_ humidified at 80 °C (100% RH) were supplied to the anode and cathode compartments with a flow rate of 1000 sccm. The durability test was conducted with the flow rate of H_2_ and O_2_ being 300 and 500 sccm under otherwise identical conditions.

### Density functional theory calculations

The density functional theory (DFT) calculations were carried out using the Vienna Ab-initio Simulation Package (VASP)^71,72^ with the projector augmented wave (PAW) method^73^ and the Perdew-Burke-Ernzerhof (PBE)^74,75^ exchange-correlation functional. Dispersion interactions were described using the DFT-D3 method proposed by Grimme^76,77^. The hetero-structure was created by the vaspkit code^78^. A 1 × 1 × 1 k-point grid with a cutoff energy of 450 eV was used for the slab optimization and the density of states (DOS) calculation. Convergence was reached when the change in energy per electron step was less than 1 × 10^−5^ eV. The conjugate gradient algorithm was used to relax all optimizable atoms until the force applied on them was less than 0.02 eV Å^−1^ for the slabs.

The anatase TiO_2_ (100) surface and Ni_4_Mo (211) surface were chosen for a better lattice match to create the hetero-structure using the slab model with a vacuum layer along the z-axis set to 15 Å. Two layers of TiO_2_ (100) $(2\sqrt{5}\times \sqrt{7})$ surface were used as the substrate, and frozen during the optimization procedure, while the Ni_4_Mo (211) $(\sqrt{10}\times 3)$ surface with a thickness of 6.15 Å above the TiO_2_ substrate was cut by $\frac{1}{3}$ along the y-axis to expose the interface of the hetero-structure, which was allowed for optimization during the whole calculation. The lattice mismatch for this hetero-structure was kept as low as 0.011%.

Adsorbates were added on the interface of the hetero-structure. The binding energy of H atom and OH species was calculated using the flowing formula:

6 $${E}_{{{{{\rm{H}}}}} \, {{{{\rm{adsorb}}}}}}={E}_{{{{{\rm{slab}}}}}+{{{{\rm{H}}}}}}-{E}_{{{{{\rm{slab}}}}}}-\frac{1}{2}{E}_{{{{{\rm{H}}}}}2}$$

7 $${E}_{{{{{\rm{OH}}}}} \, {{{{\rm{adsorb}}}}}}={E}_{{{{{\rm{slab}}}}}+{{{{\rm{OH}}}}}}{-E}_{{{{{\rm{slab}}}}}}-{E}_{{{{{\rm{OH}}}}}}$$

Where ${E}_{{{{{\rm{H}}}}} \, {{{{\rm{adsorb}}}}}}$ and ${E}_{{{{{\rm{OH}}}}} \, {{{{\rm{adsorb}}}}}}$ are the adsorption energies of H atom and OH species, ${E}_{{{{{\rm{slab}}}}}+{{{{\rm{H}}}}}}$ and ${E}_{{{{{\rm{slab}}}}}+{{{{\rm{OH}}}}}}$ are the total energies of the compositions, ${E}_{{{{{\rm{H}}}}}2}$ and ${E}_{{{{{\rm{OH}}}}}}$ are the energies of hydrogen molecule and OH species, and ${E}_{{{{{\rm{slab}}}}}}$ is the energy of the slab.

### Supplementary information

Supplementary Information

Peer Review File

## Data Availability

All data in the article and supplementary information are available from the corresponding authors upon request.
