# Supplementary material for: Metal-support interaction boosts the stability of Ni-based electrocatalysts for alkaline hydrogen oxidation
Source: Nat Commun. 2024 Jan 2;15:76. doi: 10.1038/s41467-023-44320-w (PMC10762024; doi:10.1038/s41467-023-44320-w)
Supplement: Supplementary file 1 — Supplementary Information [file 41467_2023_44320_MOESM1_ESM.pdf]

Supplementary Information

Metal-support Interaction Boosts the Stability of Ni-based Electrocatalysts for Alkaline Hydrogen Oxidation

Xiaoyu Tian,[1$] Renjie Ren,[2$] Fengyuan Wei,[2] Jiajing Pei,[3] Zhongbin Zhuang,[4] Lin Zhuang[2]* and Wenchao Sheng[1]*

Affiliations:

1 State Key Laboratory of Pollution Control and Resource Reuse,

College of Environmental Science and Engineering, Tongji University,

Shanghai Institute of Pollution Control and Ecological Security, Shanghai 200092, P.R. China

2 College of Chemistry and Molecular Sciences,

Hubei Key Laboratory of Electrochemical Power Sources, Wuhan University, Wuhan 430072, P.R. China

3 Beijing Synchrotron Radiation Facility, Institute of High Energy Physics, Chinese Academy of Sciences, Beijing 100049, P.R. China

4 State Key Laboratory of Organic-Inorganic Composites, Beijing University of Chemical Technology, Beijing 100029, P.R. China

$ These authors contribute equally

* Corresponding authors: wsheng@tongji.edu.cn, lzhuang@whu.edu.cn

Supplementary figures S1 to S39

Supplementary tables S1 to S7

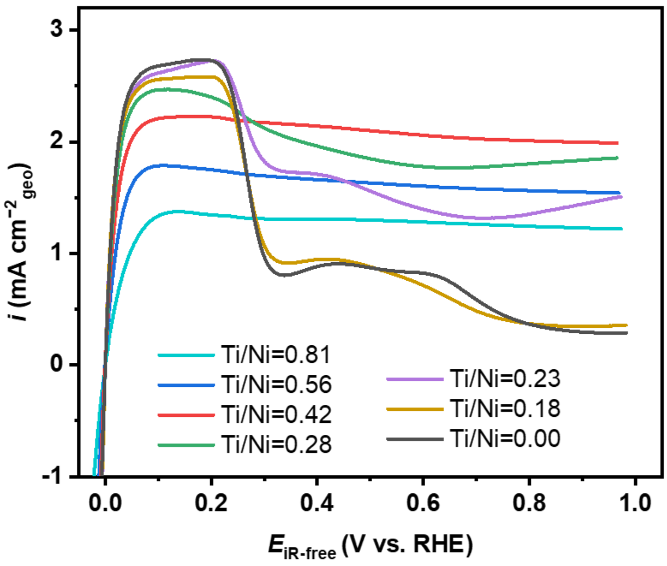

Figure S1. Positive-going sweeps of the HOR polarization curves of Ni_4Mo/TiO_2 with various Ti/Ni ratios in H_2-saturated 0.1 M NaOH at 1600 r.p.m with a scanning rate of 0.5 mV s^{-1}. The potentials are *iR*-corrected. The Ni loadings are 358, 393, 376, 409, 397, 380 and 477 μg_{Ni} cm^{-2}_{geo} for Ti/Ni=0.81, 0.56, 0.42, 0.28, 0.23, 0.18 and 0.00, respectively.

Table S1. Ti/Ni molar ratios, Ni/Mo molar ratios, Ni loadings and mass activities of Ni_4Mo/TiO_2 with various Ti/Ni ratios.

Ti/Ni[a]	Ni/Mo[a]	Loading (μg_{Ni} cm^{-2}_{geo})	Mass activity (A g^{-1}_{Ni})
0	3.8	477	9.6±0.5
0.18	3.9	380	13.4±0.3
0.23	3.8	397	12.6±0.3
0.28	3.9	409	10.4±0.5
0.42	3.8	376	10.1±0.9
0.56	3.9	393	4.8±1.5
0.81	3.9	358	3.8±0.2

Note 1: a is the molar ratio.

Note 2: The error bars are standard deviations of at least three sets of experimental repeats.

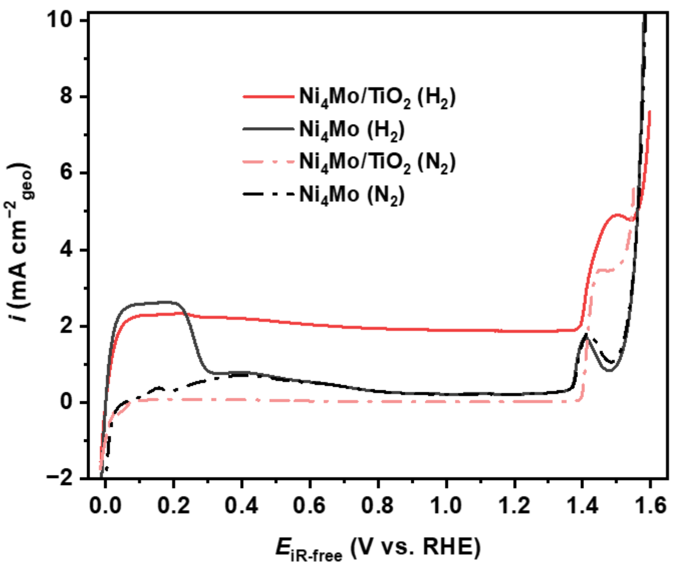

Figure S2. Positive-going sweeps of the cyclic voltammograms of Ni_4Mo and Ni_4Mo/TiO_2 in H_2 and N_2-saturated 0.1 M NaOH at 1600 r.p.m with a scanning rate of 0.5 mV s^{-1}. The potentials are *iR*-corrected. The Ni loadings are 477 and 376 μg_{Ni} cm^{-2}_{geo} for Ni_4Mo and Ni_4Mo/TiO_2.

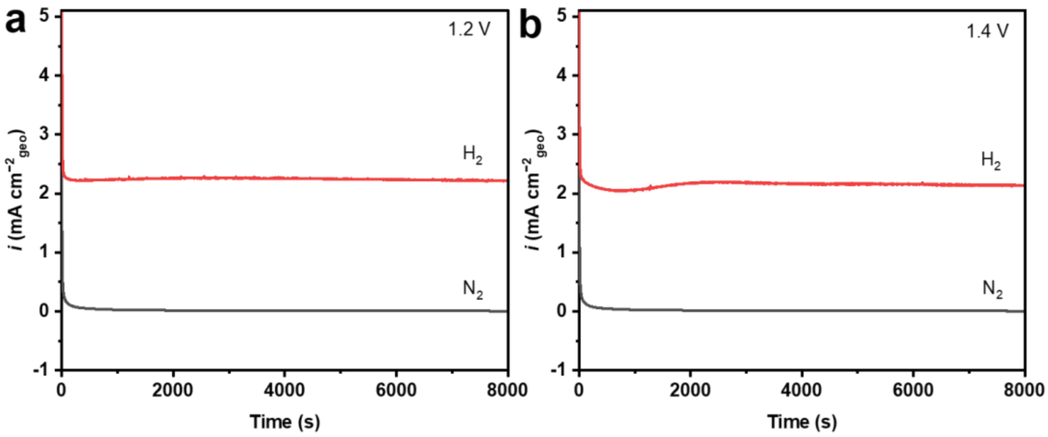

Figure S3. Chronoamperometry curves of Ni_4Mo/TiO_2 at (a) 1.2 V and (b) 1.4 V in H_2 and N_2-saturated 0.1 M NaOH at 1600 r.p.m. The potentials are not *iR*-corrected. The Ni loading is 376 μg_{Ni} $cm^{-2}{}_{geo}$ for Ni_4Mo/TiO_2.

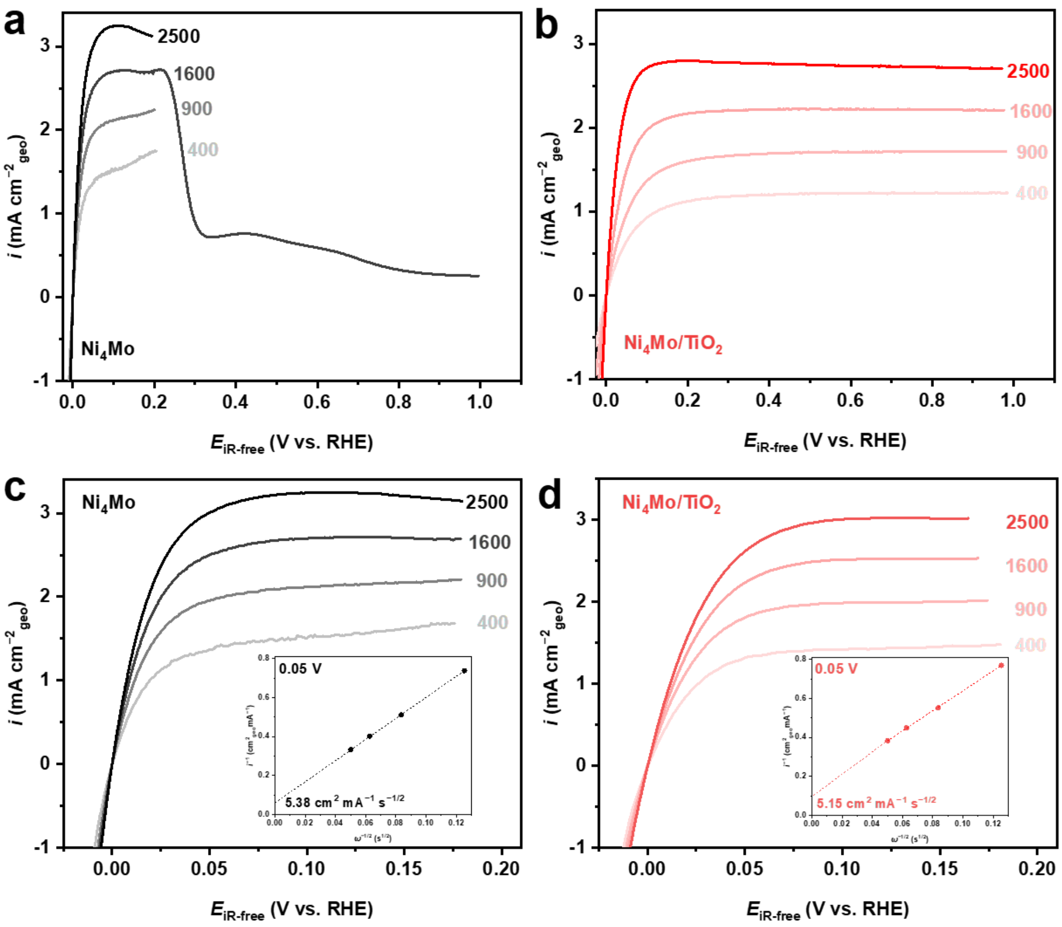

Figure S4. Positive-going sweeps of the HOR polarization curves of (a, c) Ni_4Mo and (b, d) Ni_4Mo/TiO_2 recorded in H_2-saturated 0.1 M NaOH at various rotation speeds with a scanning rate of 0.5 mV s^{-1}. The insets show the Koutecky-Levich plots at 0.05 V. The potentials are *iR*-corrected. The Ni loadings are 477 and 376 μg_{Ni} cm^{-2}_{geo} for Ni_4Mo and Ni_4Mo/TiO_2.

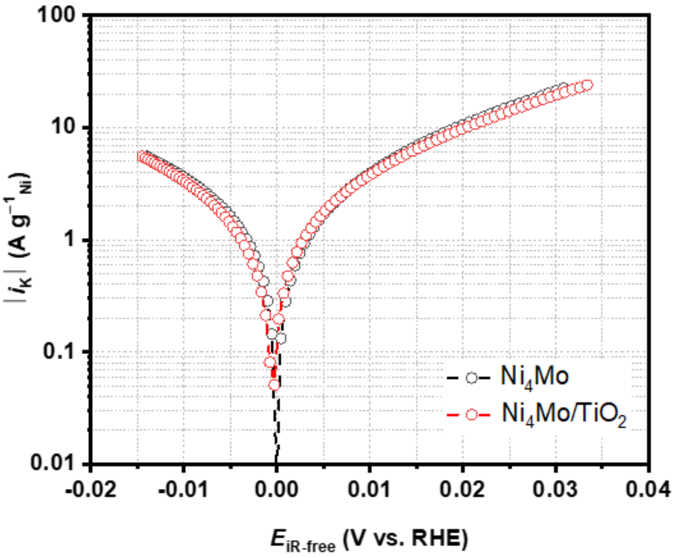

Figure S5. Kinetic currents of HER/HOR on Ni_4Mo and Ni_4Mo/TiO_2 in H_2-saturated 0.1M NaOH at 1600 r.p.m. The potentials are *iR*-corrected. The Ni loadings are 477 and 376 μg_{Ni} cm^{-2}_{geo} for Ni_4Mo and Ni_4Mo/TiO_2.

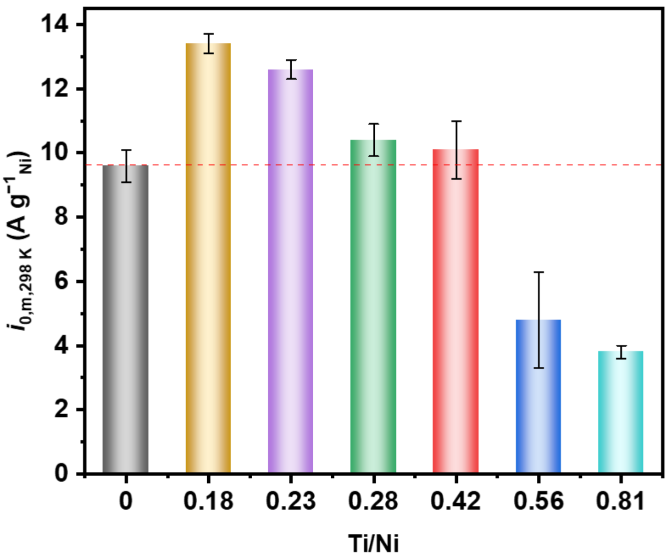

Figure S6. Mass activities of Ni_4Mo/TiO_2 with various Ti/Ni ratios. The error bars are standard deviations of at least three sets of experimental repeats. The Ni loadings are 358, 393, 376, 409, 397, 380 and 477 μg_{Ni} $cm^{-2}{}_{geo}$ for Ti/Ni=0.81, 0.56, 0.42, 0.28, 0.23, 0.18 and 0.00, respectively.

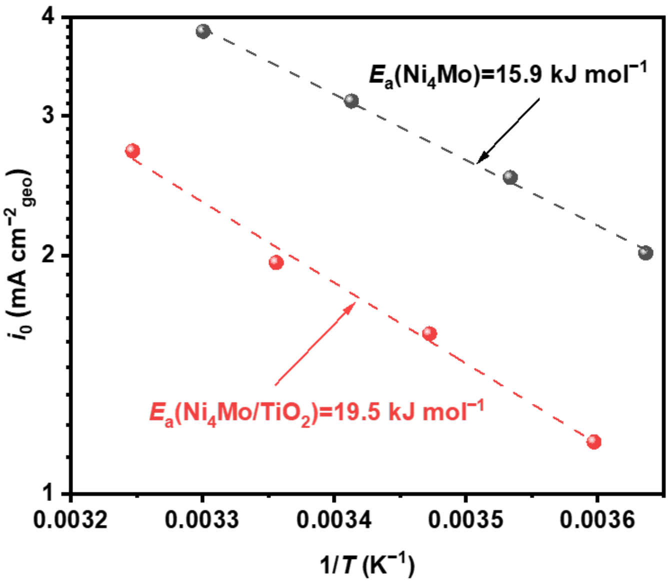

Figure S7. Arrhenius plots of the HER/HOR exchange current densities on Ni_4Mo and Ni_4Mo/TiO_2 in 0.1 M NaOH. The Ni loadings are 477 and 376 μg_{Ni} $cm^{-2}{}_{geo}$ for Ni_4Mo and Ni_4Mo/TiO_2.

Table S2. Experimental parameters and deactivation potentials of Ni-based non-precious metal electrocatalysts for the alkaline HOR.

Material	Loading ($\mu g\ cm^{-2}$)	Mass activity ($A\ g^{-1}$)	Mass activity at 0.05 V ($A\ g^{-1}$)	Scan rate ($mV\ s^{-1}$)	Deactivation potential (V)	Electrolyte	Ref.
Ni_4Mo/TiO_2	376(Ni)<br>538(Ni+Mo)<br>874(cat)	10.1(Ni)<br>7.1(Ni+Mo)<br>4.3(cat)	29.6(Ni)<br>20.7(Ni+Mo)<br>12.7(cat)	0.5	1.20	0.1M NaOH	This work
Ni_4Mo	477(Ni)<br>704(Ni+Mo)	9.6(Ni)<br>6.5(Ni+Mo)	26.2(Ni)<br>17.8(Ni+Mo)	0.5	0.20		
Ni_2W/TiO_2	312(Ni)<br>751(Ni+W)<br>1060(cat)	6.8(Ni)<br>2.8(Ni+W)<br>2.0(cat)	——	0.5	1.20		
Ni_2W	349(Ni)<br>817(Ni+W)	5.7(Ni)<br>2.4(Ni+W)	——	0.5	0.23		
NiMo/KB	100(M+C)	4.5(M)	——	5	0.10	0.1M NaOH	1
$Ni_{0.95}Cu_{0.05}/C$	25(Ni+Cu)	2.5	——	2	0.15	0.1M NaOH	2
Ni/N-CNT	250(Ni)	3.5(Ni)	9.3(Ni)	1	Up to 0.08	0.1M KOH	3
CoNiMo	410(Ni)	5.0(Ni)	14.7(Ni)	Steady state	0.10	0.1 M KOH	4
Ni@h-BN	250(Ni)	3.5(Ni)	——	5	Up to 0.13	0.1M NaOH	5
Ni@C	100(Ni)	4.5(Ni)	——	10	Up to 0.10	0.1 M KOH	6
Ni_3N/C	160(Ni_3N)	12.0(cat)	24(cat)	1	0.26	0.1 M KOH	7
Ni/NiO/C	500 (cat)	——	5	1	Up to 0.10	0.1 M KOH	8
Ni/SC	138 (Ni)	7.4(Ni)	8.6(Ni)	5	Up to 0.10	0.1 M KOH	9
Ni/NC	167 (NI)	4.8(Ni)	4.8(Ni)	5	Up to 0.08	0.1 M KOH	9
Ni/BC	168(Ni)	2.0(Ni)	2.2(Ni)	5	Up to 0.10	0.1 M KOH	9
np-Ni_3N	160(metal)	10.3	30	1	Up to 0.15	0.1 M KOH	10
Ni_3B/Ni	142(Ni)	7.0(Ni)	25(Ni)	5	Up to 0.11	0.1 M KOH	11
Ni_4Mo	200 (Ni+Mo)	14.1	54	1	0.20	0.1 M KOH	12
$Ni_3N/Ni/NF$	——	——	——	Steady state	Up to 0.10	0.1 M KOH	13
Ni_4Mo	500 (Ni+Mo)	6.8(metal)	68(metal)	0.5	Up to 0.20	0.1 M KOH	14
Ni_4W	500 (Ni+W)	3.7(metal)	17(metal)	0.5	Up to 0.20	0.1 M KOH	14
Ni/MoO_2	765(cat)	——	——	5	Up to 0.10	0.1 M KOH	15
CeO_2/Ni	14(Ni)	7.6(Ni)	12(Ni)	5	Up to 0.11	0.1 M KOH	16
PS-MoNi	——	——	——	1	0.32	0.1 M KOH	17
Ni/MoO_2	765(cat)	9.8(Ni)	39(Ni)	1	Up to 0.20	0.1 M KOH	18
$Ni_{5.2}WCu_{2.2}$	9200(cat)	2.5(Ni)	2.6(Ni)	1	0.30	0.1 M KOH	19
Ni/Ni_3N-C	166(Ni)	5.2(Ni)	12(Ni)	5	Up to 0.15	0.1 M KOH	20
4.3%N-Ni	320(cat)	9.3(cat)	77(cat)	1	0.25	0.1 M KOH	21
Ni@CN_x	500(cat)	——	1.2(Ni)	5	0.16	0.1 M KOH	22
Ni@Oi-Ni	142(Ni)	——	86(Ni)	5	0.26	0.1 M KOH	23
$Ni_{52}Mo_{13}Nb_{35}$	8000 (metal)	——	——	1	0.80	0.1 M KOH	24

Table S3. Experimental parameters and AEMFC performances of non-precious metal anode electrocatalysts.

**Anode catalyst**	**Cathode catalyst**	**Cell temperature (ºC)**	**Membrane**	**Backpressure (kpa)**	**Peak power density (mW cm^{-2})**	**Durability**	**Ref.**
Ni_4Mo/TiO_2 1.35 mg_{Ni} cm^{-2}	Pt/C 0.4 mg_{Pt} cm^{-2}	80	QAPPT	200	520	400 mA cm^{-2} for 100 h (0.74-0.50 V)	This work
Ni_4Mo 1.35 mg_{Ni} cm^{-2}	Pt/C 0.4 mg_{Pt} cm^{-2}	80	QAPPT	200	188	——	This work
Ni-H_2-NH_3 6.4 mg_{Ni} cm^{-2}	Pt/C 0.2 mg_{Pt} cm^{-2}	95	PAP-TP-85	250	628	0.7 V for 40 h (~280 mA cm^{-2})	25
Ni-H_2-NH_3 6.4 mg_{Ni} cm^{-2}	Co-Mn spinel 1.2 mg_{cat} cm^{-2}	95	PAP-TP-85	250	450	——	25
Ni@CN_x 15 mg_{Ni} cm^{-2}	Pt/C 0.4 mg_{Pt} cm^{-2}	80	QAPPT	200	480	200 mA cm^{-2} for 100 h (~0.7 V)	22
Ni@CN_x 15 mg_{Ni} cm^{-2}	$MnCo_2O_4$ 1.5 mg_{cat} cm^{-2}	80	QAPPT	200	210	——	22
$Ni_{52}Mo_{13}Nb_{35}$ 4 mg_{cat} cm^{-2}	Pt/C 0.4 mg_{Pt} cm^{-2}	90	Alkymer W-25	200	390	200 mA cm^{-2} for 50 h (0.7-0.4 V)	24
NiCu/KB 4 mg_{cat} cm^{-2}	Pd/C 0.20 mg_{pd} cm^{-2}	80	Tokuyama A201	138	350	——	26
Ni@Oi-Ni 1.3 mg_{Ni} cm^{-2}	Pt/C 0.4 mg_{Pt} cm^{-2}	80	QAPPT	200	274	——	23
Ni/Ni_3N-C 0.5 mg_{metal} cm^{-2}	Pt/C 0.2 mg_{Pt} cm^{-2}	80	QAPPT	200	223	——	20
Ni@C 5.0 mg_{Ni} cm^{-2}	Pt/C 0.4 mg_{Pt} cm^{-2}	80	QAPPT	200	160	0.7 V (~70 mA cm^{-2}) for 120 h	6
NiMo/KB 4.0 mg_{cat} cm^{-2}	Pd/C 0.2 mg_{pd} cm^{-2}	70	Tokuyama A201	138	120	——	1
Ni/C 5.0 mg_{Ni} cm^{-2}	Ag/C 0.5 mg_{Ag} cm^{-2}	70	TPQPOH 152	250	76	——	27
NiCr 5.0 mg_{Ni} cm^{-2}	Ag/C 1.0 mg_{Ag} cm^{-2}	60	QAPS	130	50	——	28
NiW 17.5 mg_{cat} cm^{-2}	CoPPY/C 2.0 mg_{cat} cm^{-2}	60	xQAPS	0	40	——	29
NiCo 5.0 mg_{cat} cm^{-2}	Co_3O_4 3.0 mg_{cat} cm^{-2}	70	AT-1	——	22	——	30

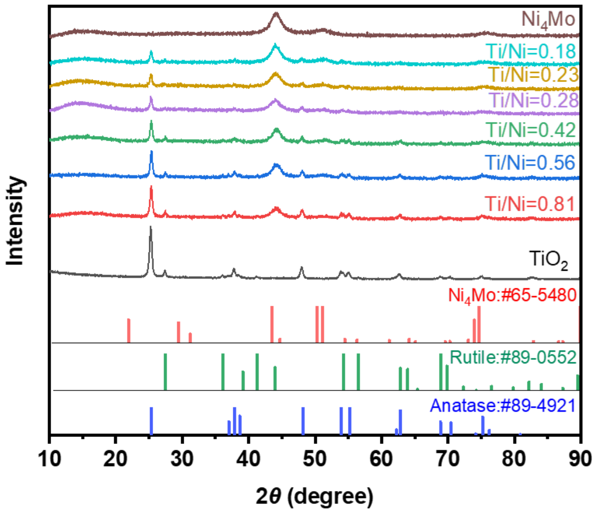

Figure S8. XRD patterns of TiO_2, Ni_4Mo and Ni_4Mo/TiO_2 with various Ti/Ni ratios. The Ni_4Mo/TiO_2 catalysts with different Ti/Ni ratios display the crystallographic features of both Ni_4Mo (JCPDS 65-5480) and TiO_2 (Anatase JCPDS 89-4921; Rutile phase JCPDS 89-0552). Increasing the Ti/Ni ratio leads to the decreased relative peak intensity of Ni_4Mo to TiO_2.

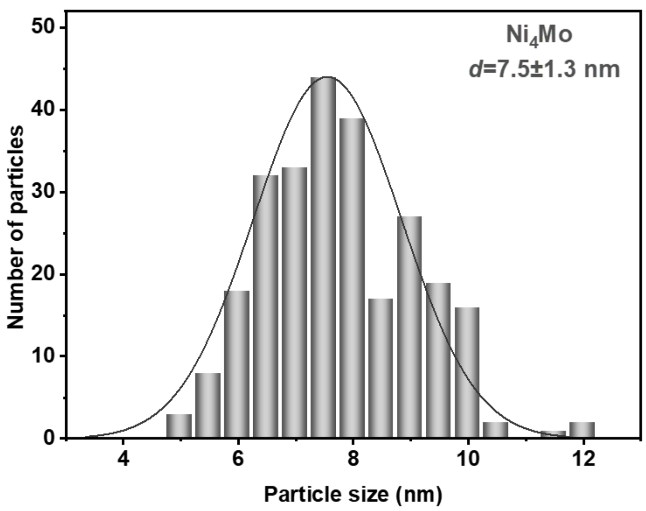

Figure S9. The particle size distribution of the Ni_4Mo catalyst. The error bar is the standard deviation of the particle size based on at least 200 counts.

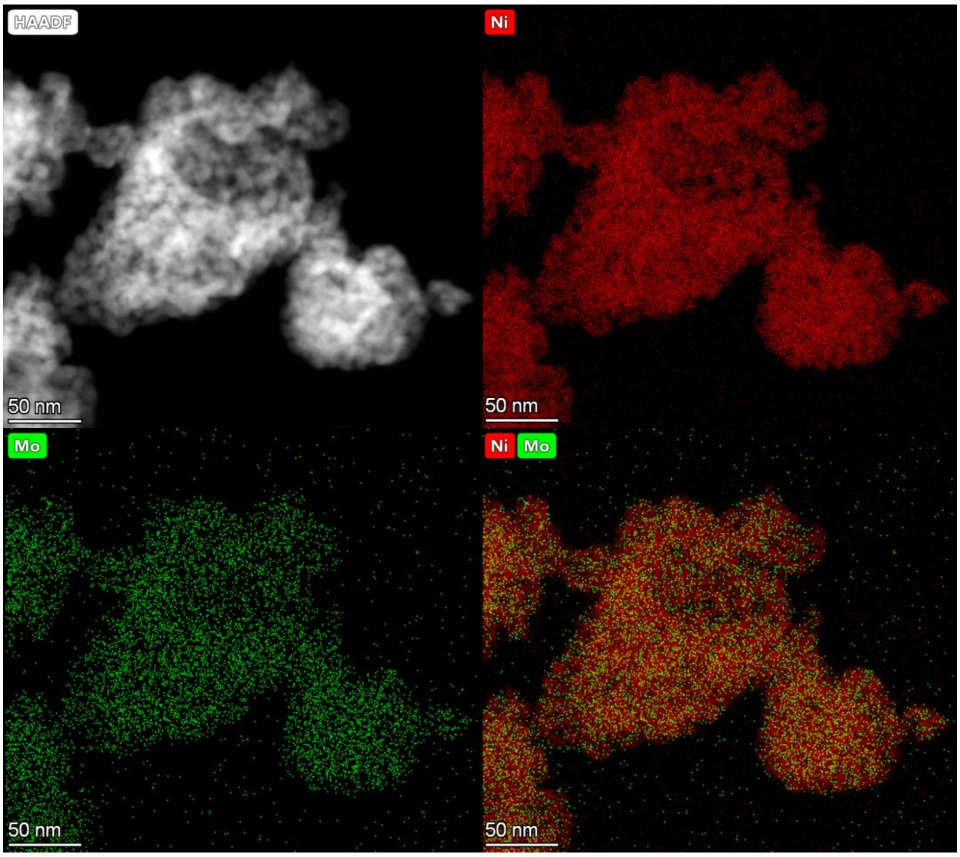

Figure S10. EDS elemental mappings of Ni and Mo of the Ni_4Mo catalyst. The Ni and Mo elements have a uniform spatial distribution, confirming the formation of a uniform Ni_4Mo alloy.

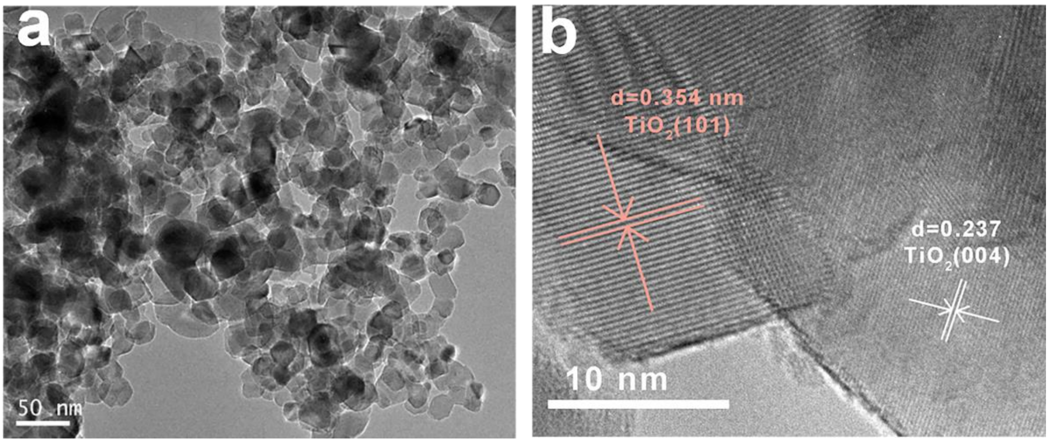

Figure S11. (a) TEM and (b) HR-TEM images of the pristine TiO_2.

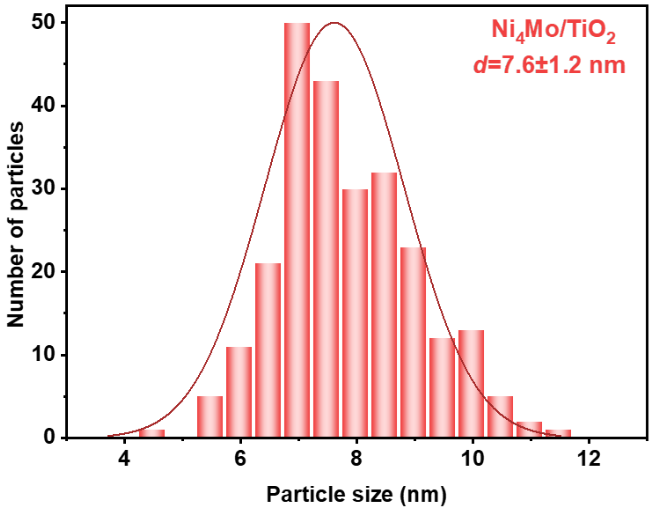

Figure S12. The Ni_4Mo particle size distribution of the Ni_4Mo/TiO_2 catalyst. The error bar is the standard deviation of the particle size based on at least 200 counts.

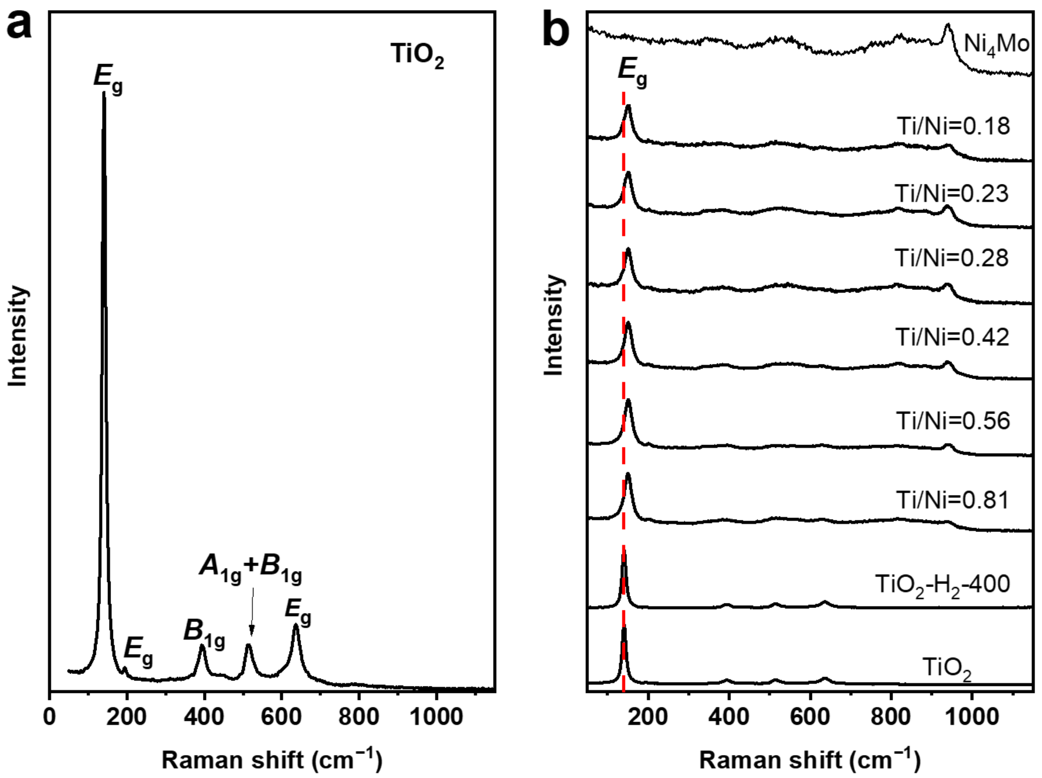

Figure S13. Raman spectra of (a) TiO_2 and (b) TiO_2-H_2-400, Ni_4Mo and Ni_4Mo/TiO_2 with various Ti/Ni ratios.

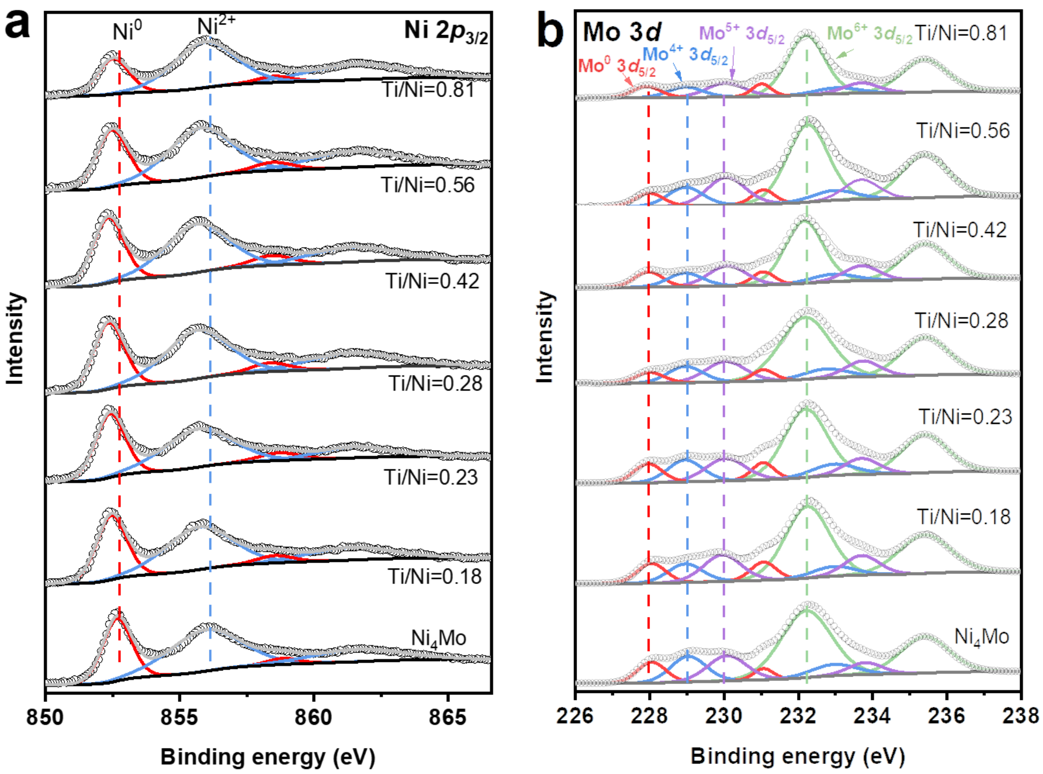

Figure S14. (a) Ni 2$p_{3/2}$ and (b) Mo 3d level XPS spectra of Ni_4Mo and Ni_4Mo/TiO_2 with various Ti/Ni ratios.

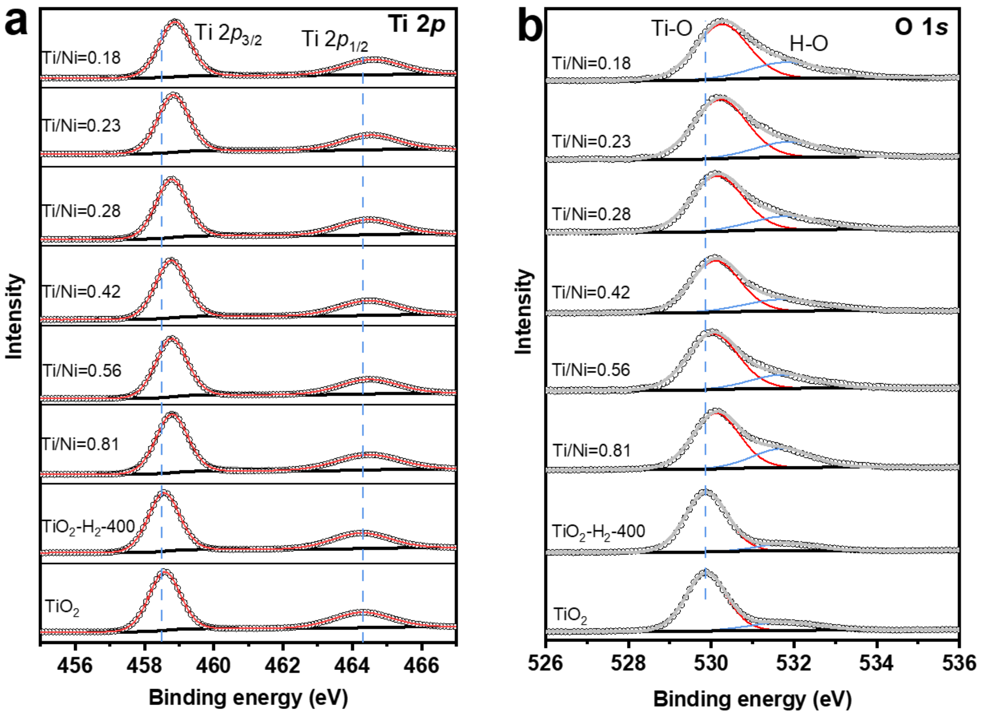

Figure S15. (a) Ti 2*p* and (b) O 1*s* level XPS spectra of TiO_2, TiO_2-H_2-400, Ni_4Mo and Ni_4Mo/TiO_2 with various Ti/Ni ratios.

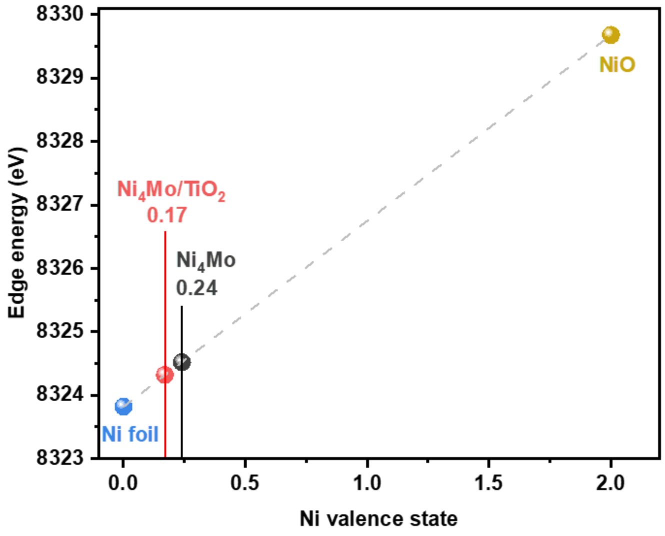

Figure S16. Relation between the Ni K-edge absorption energy and valence state of Ni, NiO, Ni_4Mo and Ni_4Mo/TiO_2.

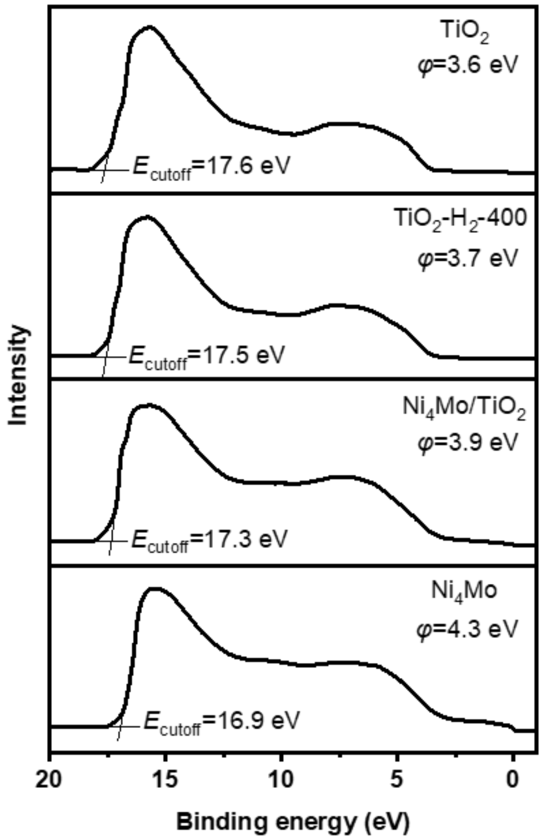

Figure S17. Valence band states of TiO_2, TiO_2-H_2-400, Ni_4Mo and Ni_4Mo/TiO_2. The UV photon energy was 21.2 eV (He I), and the sample was biased by −5 V.

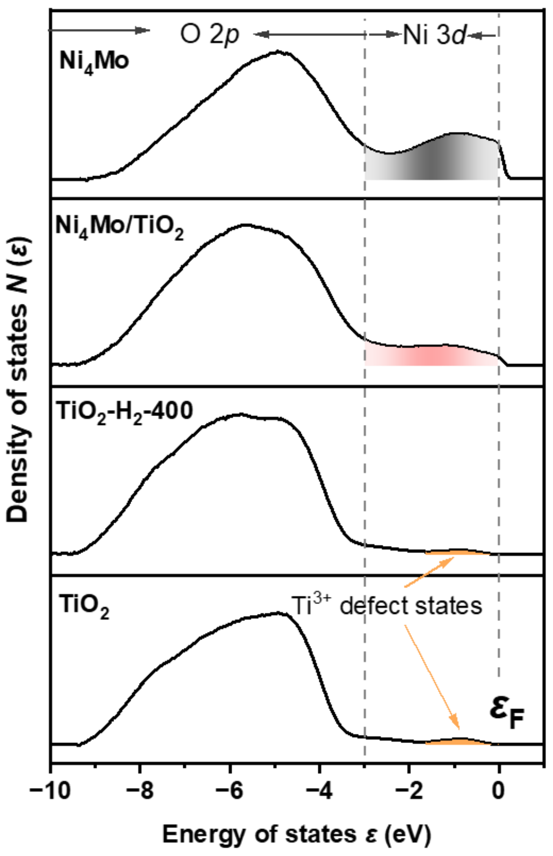

Figure S18. Density of states near the Fermi level of TiO_2, TiO_2-H_2-400, Ni_4Mo and Ni_4Mo/TiO_2 measured by UPS.

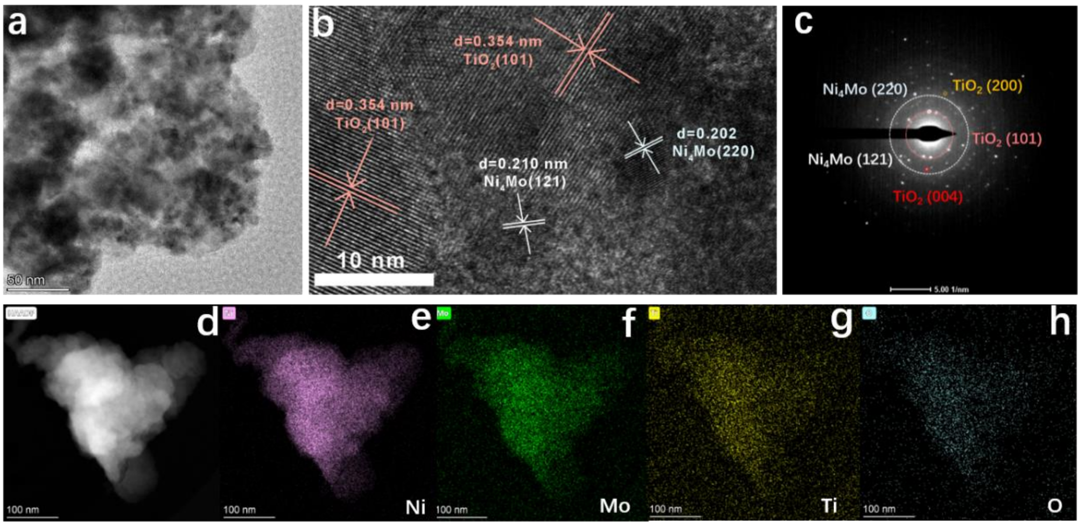

Figure S19. (a) TEM image, (b) HR-TEM image, (c) SAED pattern and (d-h) EDS elemental mappings of Ni_4Mo/TiO_2 after long-term stability test at 1.2 V (no *iR*-correction) for 2 h in H_2-saturated 0.1 M NaOH.

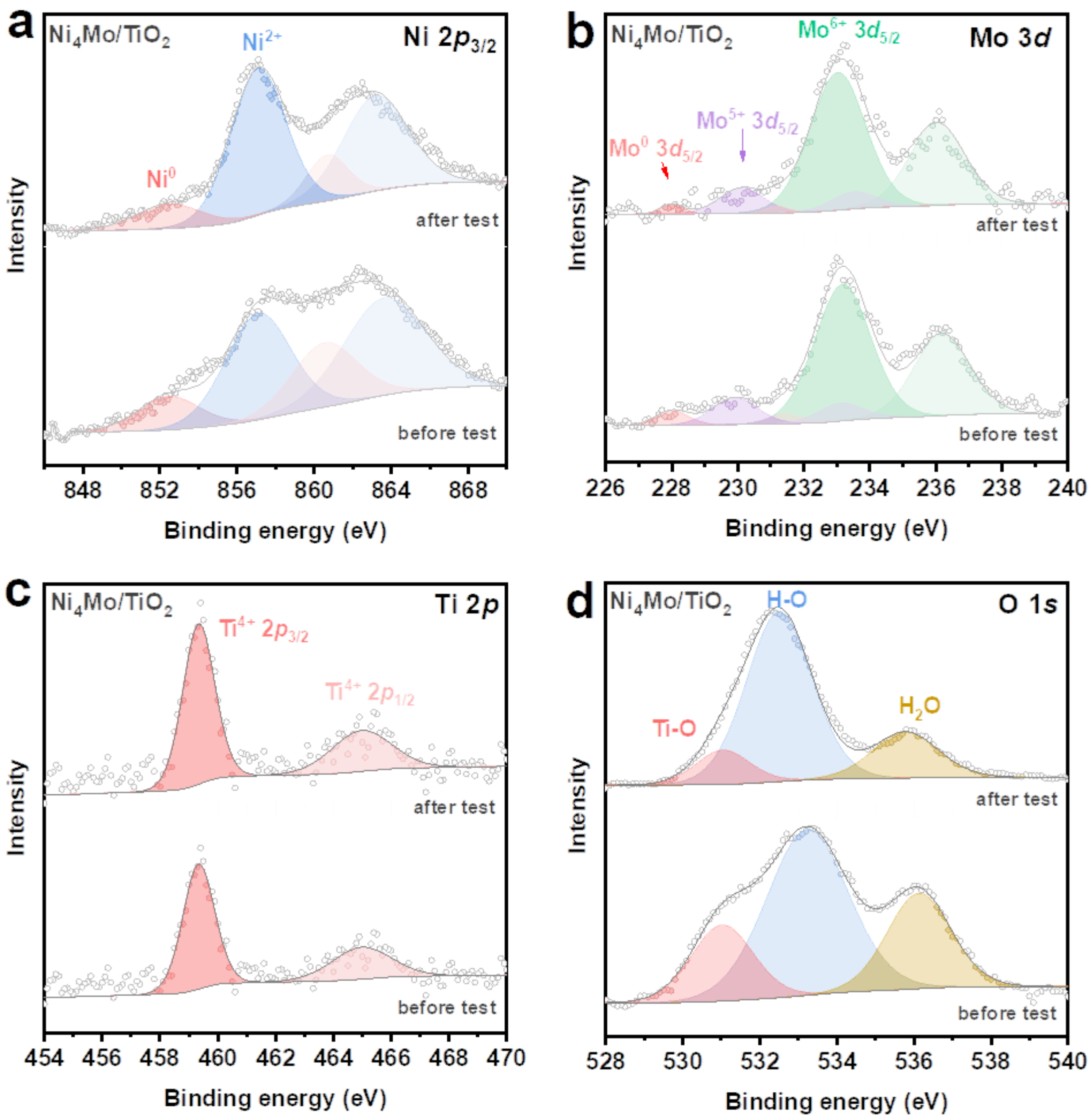

Figure S20. (a) Ni $2p_{3/2}$, (b) Mo 3*d*, (c) Ti 2*p* and (d) O 1*s* level XPS spectra of Ni_4Mo/TiO_2 before and after the long-term stability test at 1.2 V (no *iR*-correction) for 2 h in H_2-saturated 0.1 M NaOH.

Table S4. Ni/Mo molar ratios and Mo dissolution depths of Ni_4Mo and Ni_4Mo/TiO_2 before and after the CV and CA tests.

Sample	Ni/Mo			Mo dissolution depth estimated from the ICP-OES result (nm)
	XPS	EDS	ICP-OES	
Ni_4Mo	10.4	4.3	4.1	——
Ni_4Mo after CV test	20.2	6.8	6.1	0.5
Ni_4Mo after CA test	14.5	10.8	6.9	0.6
Ni_4Mo/TiO_2	7.9	4.8	4.4	——
Ni_4Mo/TiO_2 after CV test	11.6	9.1	8.1	0.8
Ni_4Mo/TiO_2 after CA test	18.5	14.2	10.6	1.1

Note: "Ni_4Mo" and "Ni_4Mo/TiO_2" samples represent the catalysts before the electrochemical measurements; "Ni_4Mo after CV test" and "Ni_4Mo/TiO_2 after CV test" samples represent the catalysts after CV tests in H_2-saturated 0.1 M NaOH from −0.05 to 1.0 V at 0.5 mV s^{-1}; "Ni_4Mo after CA test" and "Ni_4Mo/TiO_2 after CA test" samples represent the catalysts after CA tests at 1.2 V for 2 h in H_2-saturated 0.1 M NaOH. The potentials are not *iR*-corrected.

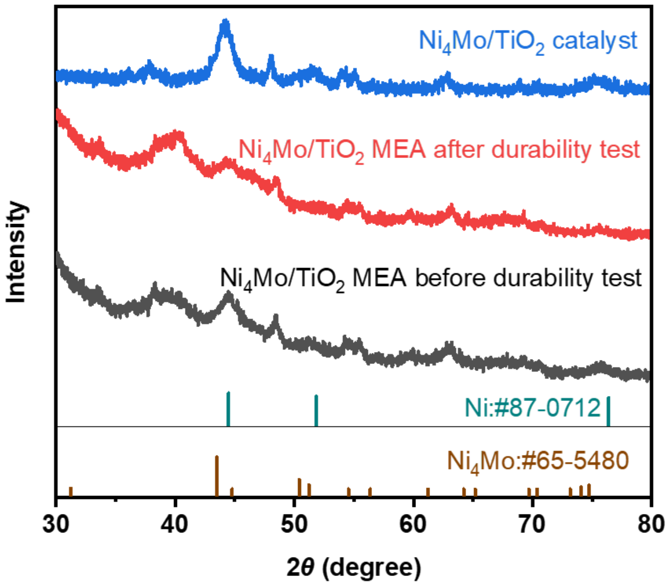

Figure S21. XRD patterns of the Ni_4Mo/TiO_2 catalyst and the MEAs before and after the durability test. The Ni_4Mo/TiO_2 MEA after the durability test exhibits negligible changes in the crystallographic structure compared to the MEA before the durability test, maintaining the Ni_4Mo phase structure (JCPDS 65-5480) as the synthesized Ni_4Mo/TiO_2 catalyst.

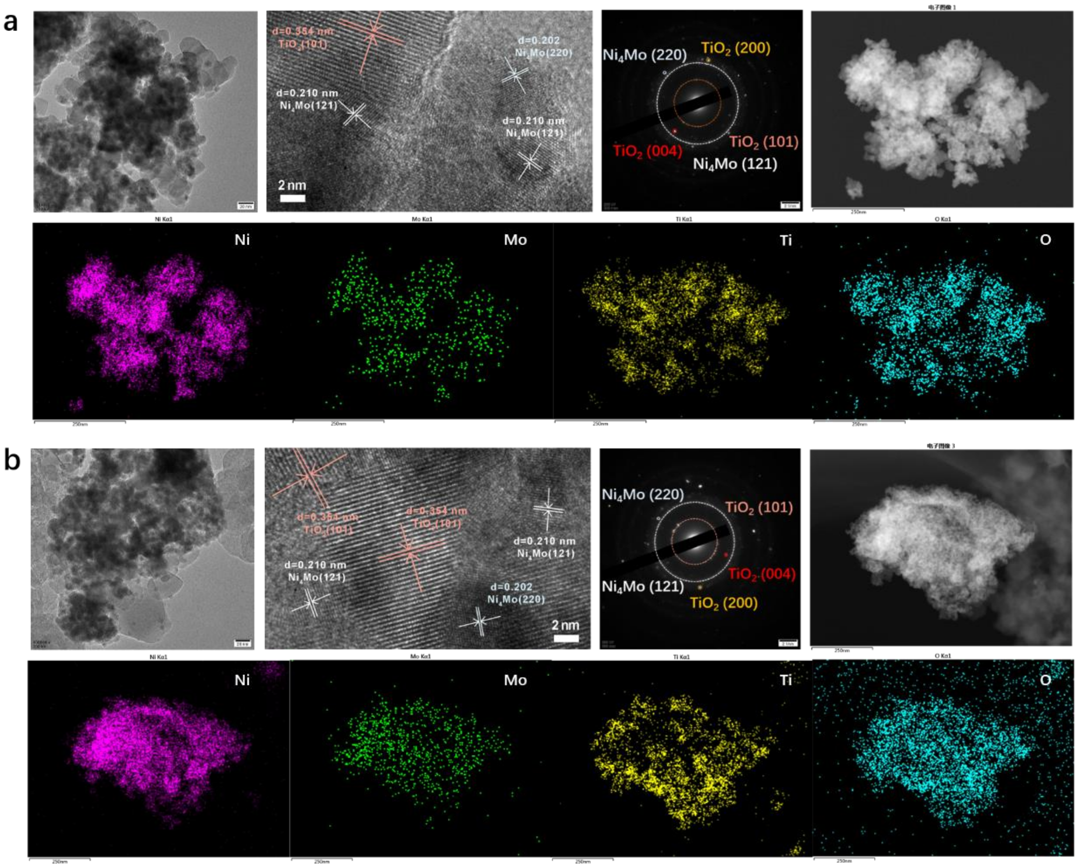

Figure S22. TEM images, HR-TEM images, SAED patterns and EDS elemental mappings of the Ni_4Mo/TiO_2 MEAs (a) before and (b) after the durability test.

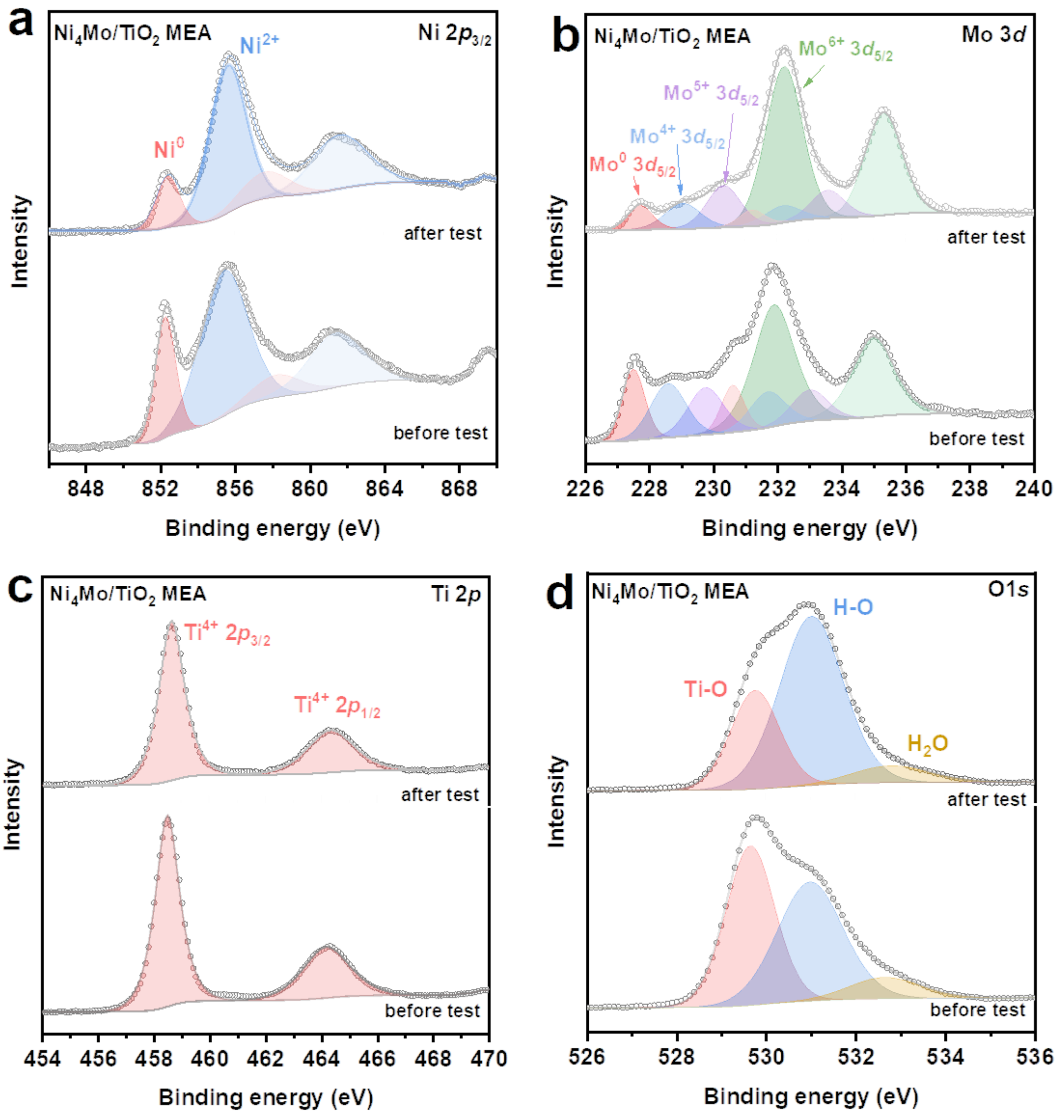

Figure S23. (a) Ni 2$p_{3/2}$, (b) Mo 3*d*, (c) Ti 2*p* and (d) O 1*s* level XPS of the Ni_4Mo/TiO_2 MEAs before and after the durability test.

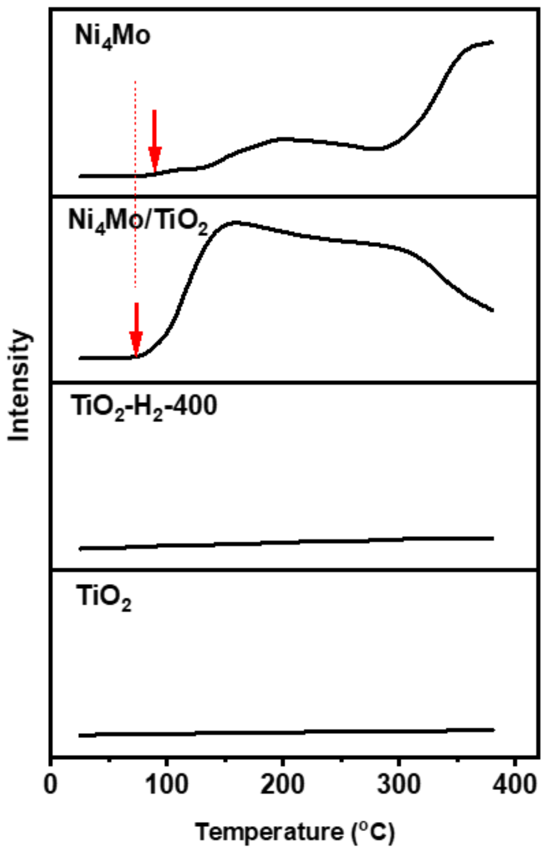

Figure S24. H_2-TPD profiles of TiO_2, TiO_2-H_2-400, Ni_4Mo and Ni_4Mo/TiO_2.The red arrows represent the hydrogen desorption temperature.

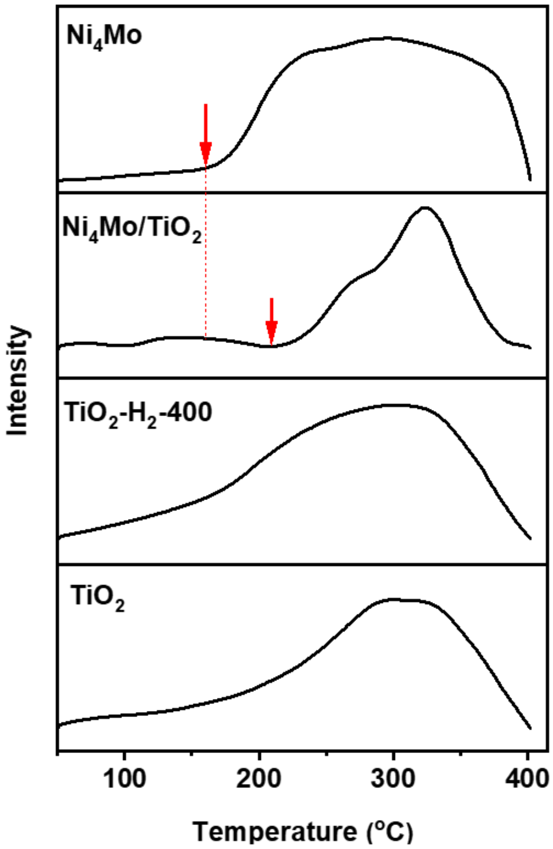

Figure S25. O_2-TPO profiles of TiO_2, TiO_2-H_2-400, Ni_4Mo and Ni_4Mo/TiO_2. The red arrows represent the catalyst oxidation temperature.

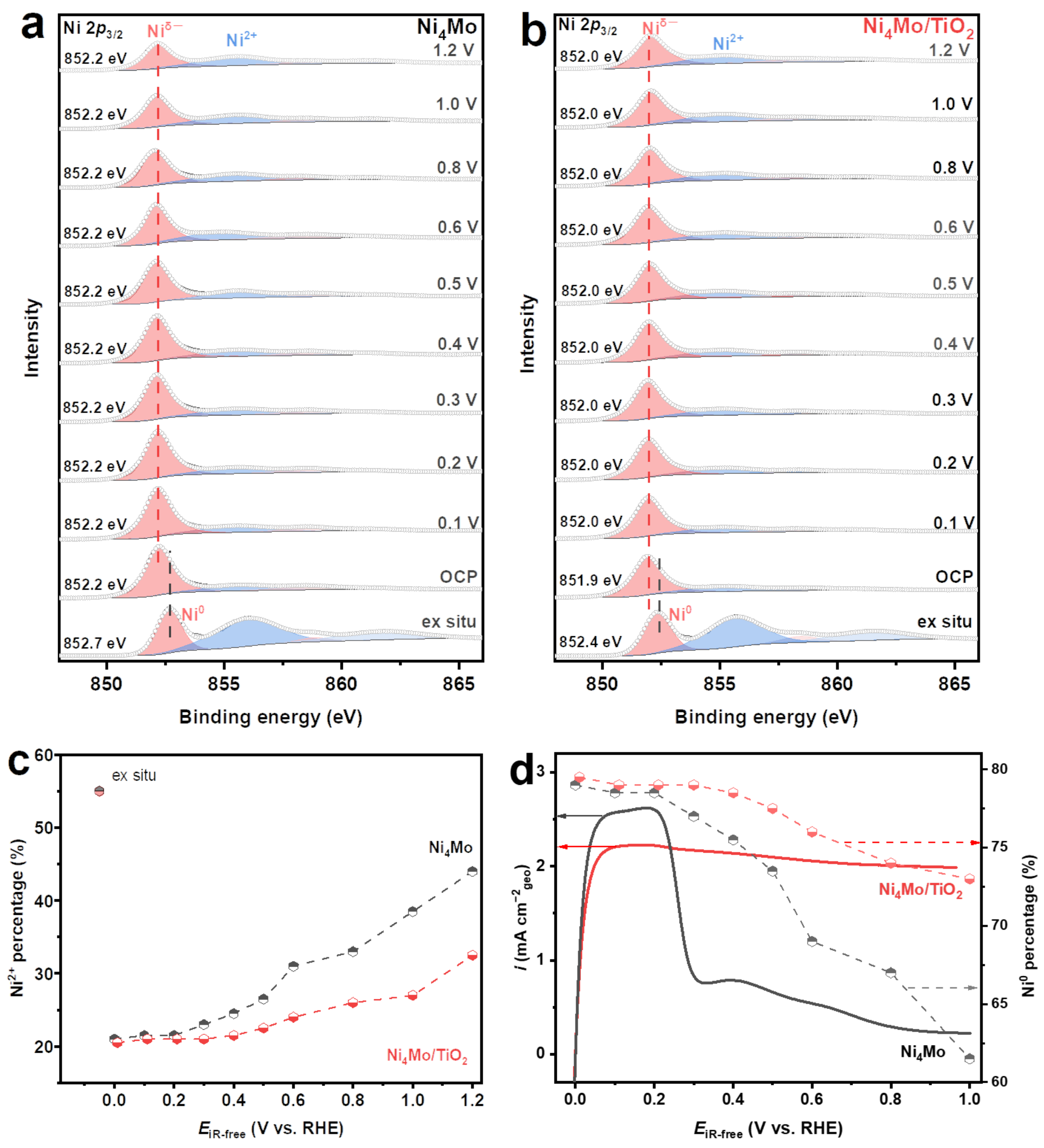

Figure S26. Quasi in situ Ni 2$p_{3/2}$ level XPS spectra of (a) Ni_4Mo and (b) Ni_4Mo/TiO_2 during the HOR at selected potentials in 0.1 M NaOH; (c) the Ni^{2+} contents estimated from XPS; and (d) the Ni^0 ($Ni^{\delta-}$) contents estimated from XPS and the HOR polarization curves. The potentials are *iR*-corrected. The Ni loadings are 477 and 376 μg_{Ni} $cm^{-2}{}_{geo}$ for Ni_4Mo and Ni_4Mo/TiO_2 in the RDE tests in (d).

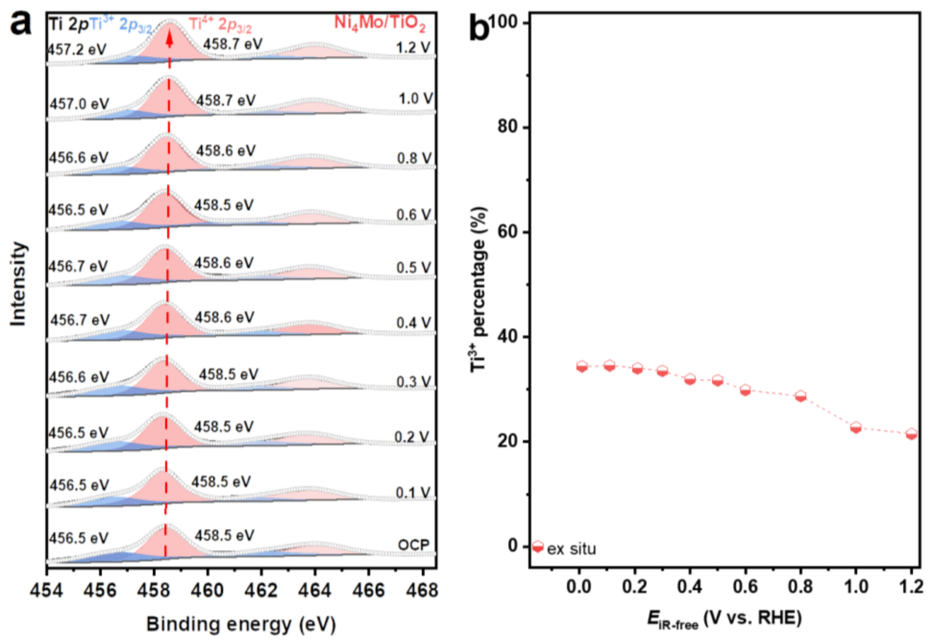

Figure S27. (a) Quasi in situ Ti 2*p* XPS spectra during the HOR at selected potentials in 0.1 M NaOH; and (b) the Ti^{3+} content estimated from XPS of Ni_4Mo/TiO_2. The potentials are *iR*-corrected.

Table S5. Vibrational modes and Raman shifts of Ni and Mo oxygenated species.

Mode	Raman shift (cm^{-1})	Ref.
Mo=O stretching mode in the MoO_4^{2-} tetrahedron	310	31
Mo=O bending mode in the MoO_4^{2-} tetrahedron	893	
bridging Mo-O-Mo symmetric stretching mode in $Mo_2O_7^{2-}$	483	31, 32
Ni-OH symmetric stretching mode of $Ni(OH)_2$	460	33-36
Mo-O stretching mode of $NiMoO_4$	939	32, 37
Ni-O bending vibration mode of γ-NiOOH	474	38-41
Ni-O stretching vibration mode of γ-NiOOH	558	

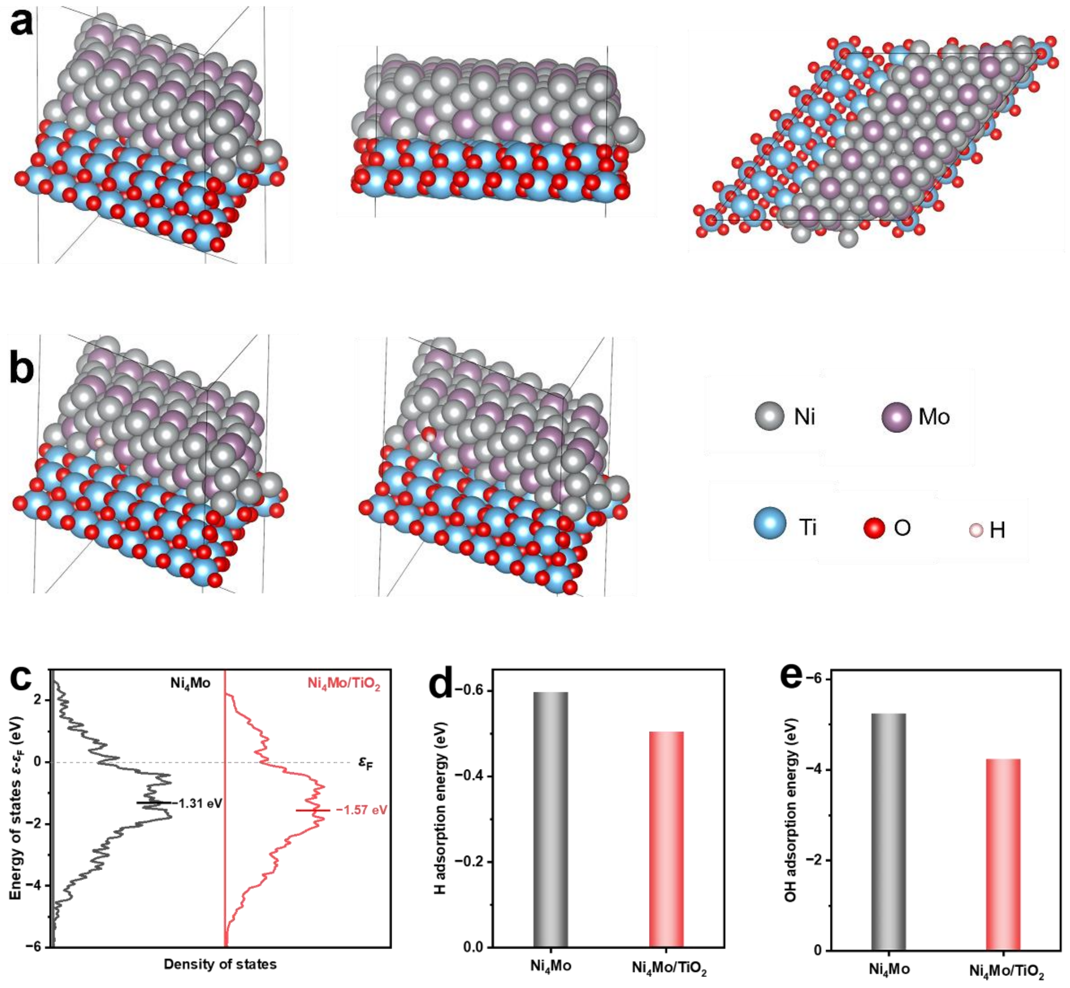

Figure S28. (a) Slab model of the Ni_4Mo/TiO_2 hetero-structure surface. 1/3 of the Ni_4Mo surface were cut off to expose the interface; (b) adsorption sites of the Ni_4Mo/TiO_2 hetero-structure surface. H atom (left) and OH species (right) were added at the interface of the hetero-structure surface; (c) density of states and *d* band center; and (d, e) adsorption energy of H atom and OH species of Ni_4Mo and Ni_4Mo/TiO_2.

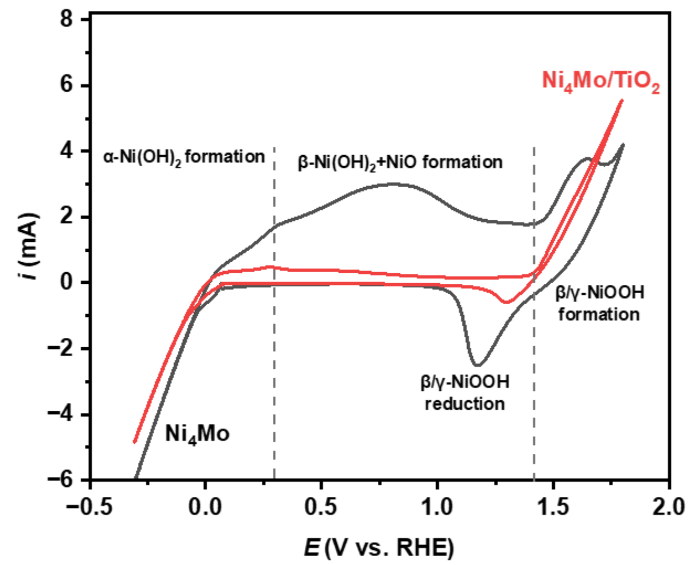

Figure S29. Cyclic voltammograms of Ni_4Mo and Ni_4Mo/TiO_2 recorded in N_2-saturated 0.1 M NaOH with a rotation speed of 1600 r.p.m and a scanning rate of 20 mV s^{-1}. The potentials are not *iR*-corrected. The Ni loadings are 376 μg_{Ni} cm^{-2}_{geo} for Ni_4Mo and Ni_4Mo/TiO_2.

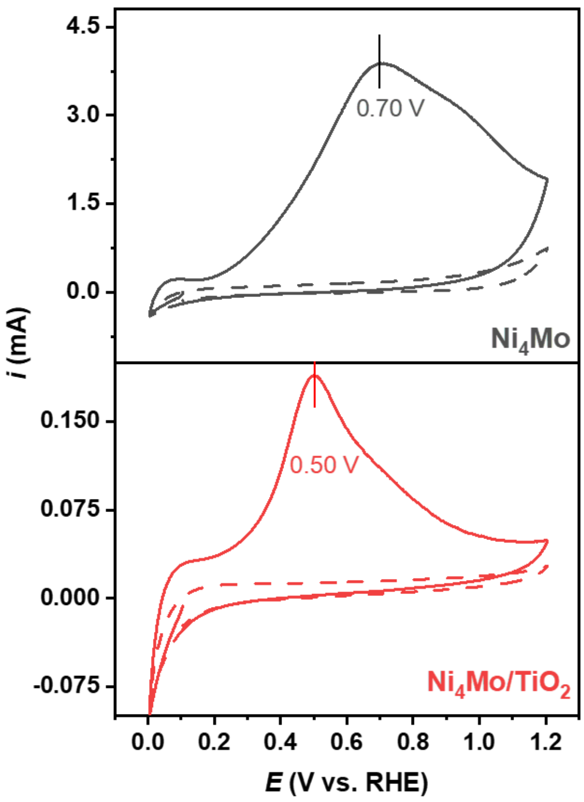

Figure S30. CO-stripping on Ni_4Mo and Ni_4Mo/TiO_2 collected in 0.1 M NaOH with a rotation speed of 1600 r.p.m and a scanning rate of 20 mV s^{-1}. The dash lines show the second cycle of the measurements. The potentials are not *iR*-corrected. The Ni loadings are 477 and 376 μg_{Ni} $cm^{-2}{}_{geo}$ for Ni_4Mo and Ni_4Mo/TiO_2.

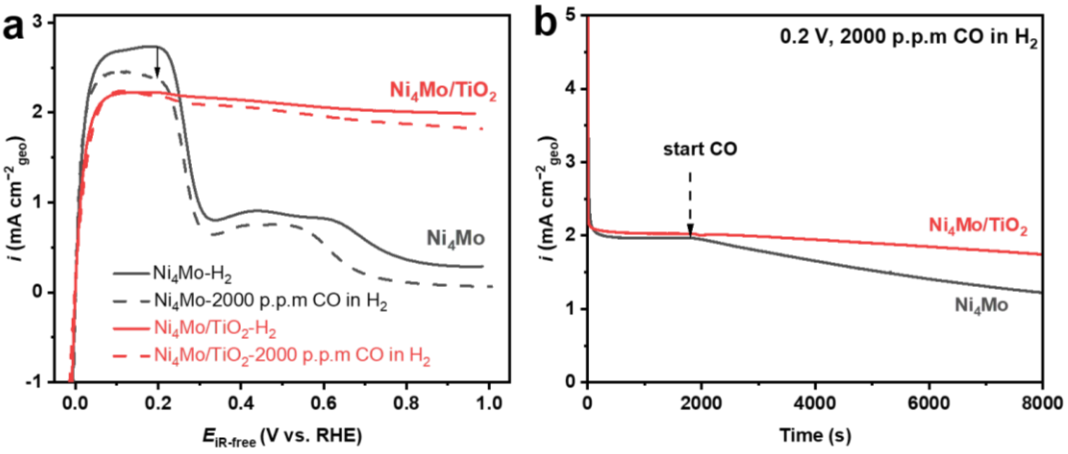

Figure S31. (a) Positive-going sweeps of the HOR polarization curves of Ni_4Mo and Ni_4Mo/TiO_2 in H_2-saturated 0.1 M NaOH at 1600 r.p.m with a scanning rate of 0.5 mV s^{-1} with/without 2000 p.p.m CO. The potentials are *iR*-corrected. (b) Chronoamperometry curves at 0.2 V for Ni_4Mo and Ni_4Mo/TiO_2 in H_2-saturated 0.1 M NaOH with 2000 p.p.m CO introduced after 30 mins. The potentials are not *iR*-corrected. The Ni loadings are 477 and 376 μg_{Ni} $cm^{-2}{}_{geo}$ for Ni_4Mo and Ni_4Mo/TiO_2.

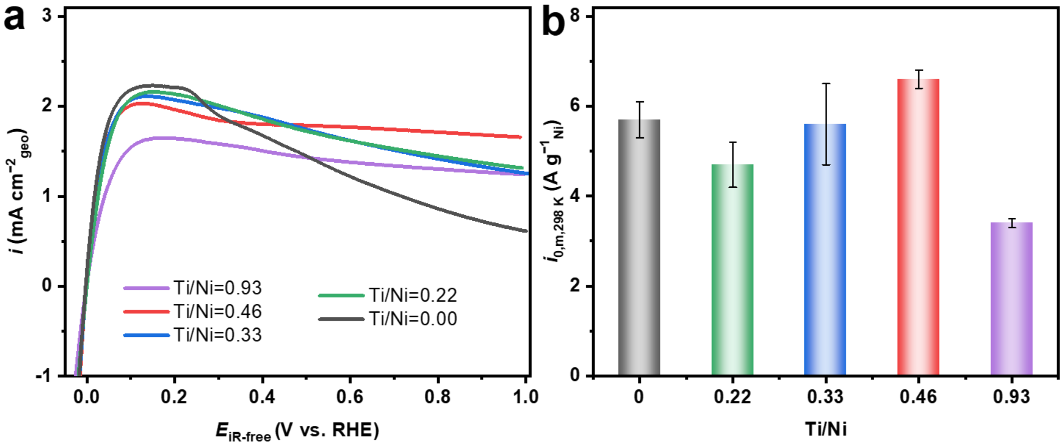

Figure S32. (a) Positive-going sweeps of the HOR polarization curves; and (b) mass activities of Ni_2W/TiO_2 with various Ti/Ni ratios in H_2-saturated 0.1 M NaOH at 1600 r.p.m with a scanning rate of 0.5 mV s^{-1}. The potentials are *iR*-corrected. The error bars are standard deviations of at least three sets of experimental repeats. The Ni loadings are 312, 312, 321, 329 and 349 μg_{Ni} $cm^{-2}{}_{geo}$ for Ti/Ni=0.93, 0.46, 0.33, 0.22 and 0.00, respectively.

Table S6. Ti/Ni molar ratios, Ni/W molar ratios, Ni loadings and mass activities of Ni_2W/TiO_2 with various Ti/Ni ratios.

Ti/Ni^a	Ni/W^a	Loading (μg_{Ni} $cm^{-2}{}_{geo}$)	Mass activity (A $g^{-1}{}_{Ni}$)
0	2.3	349	5.7±0.4
0.22	2.1	329	4.7±0.5
0.33	2.1	321	5.6±0.9
0.46	2.2	312	6.8±0.2
0.93	2.2	312	3.4±0.1

Note 1: a is the molar ratio.

Note 2: The error bars are standard deviations of at least three sets of experimental repeats.

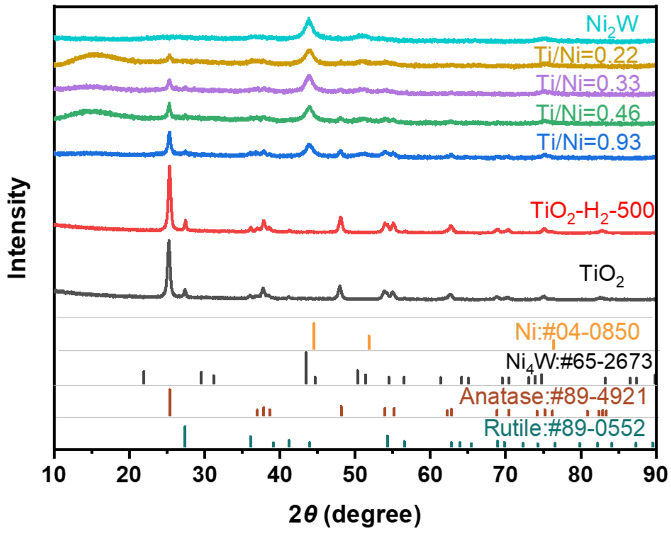

Figure S33. XRD patterns of TiO_2, TiO_2-H_2-500, Ni_2W and Ni_2W/TiO_2 with various Ti/Ni ratios. The Ni_2W catalyst shows a diffraction peak at 43.8 ^{o}, which is between the Ni (111) plane at 44.5 ^{o} and Ni_4W (211) plane at 43.5 ^{o}. The Ni_2W/TiO_2 catalysts with different Ti/Ni ratios display the crystallographic features of both Ni_2W and TiO_2. Increasing the Ti/Ni ratio leads to the decreased relative peak intensity of Ni_2W to TiO_2.

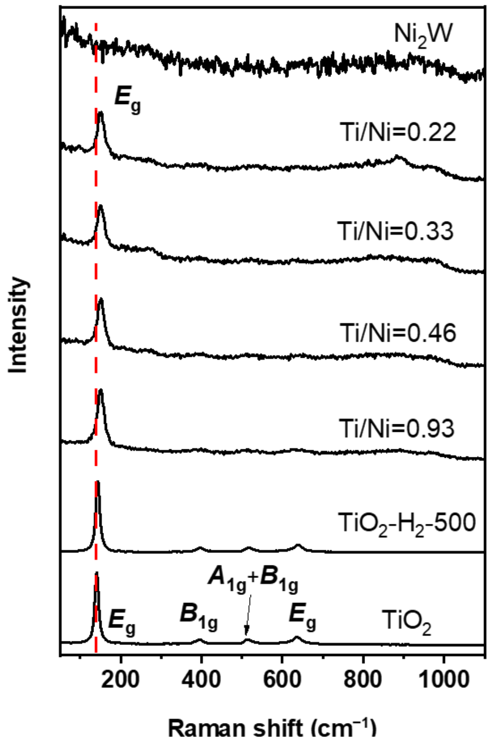

Figure S34. Raman spectra of TiO_2, TiO_2-H_2-500, Ni_2W and Ni_2W/TiO_2 with various Ti/Ni ratios. TiO_2 shows six Raman-active modes at around 140 cm^{-1} (E_g), 195 cm^{-1} (E_g), 393 cm^{-1} (B_{1g}), 511 cm^{-1} (A_{1g}+ B_{1g}) and 635 cm^{-1} (E_g). TiO_2-H_2-500 (TiO_2 treated at 500 ºC in H_2) shows no detectable changes with respect to the pristine TiO_2. Decorating Ni_2W on the TiO_2 support leads to distinct blue-shift in the vibrational mode of E_g from 140 cm^{-1} to 151 cm^{-1} and peak broadening, which is most likely due to a compressive strain.

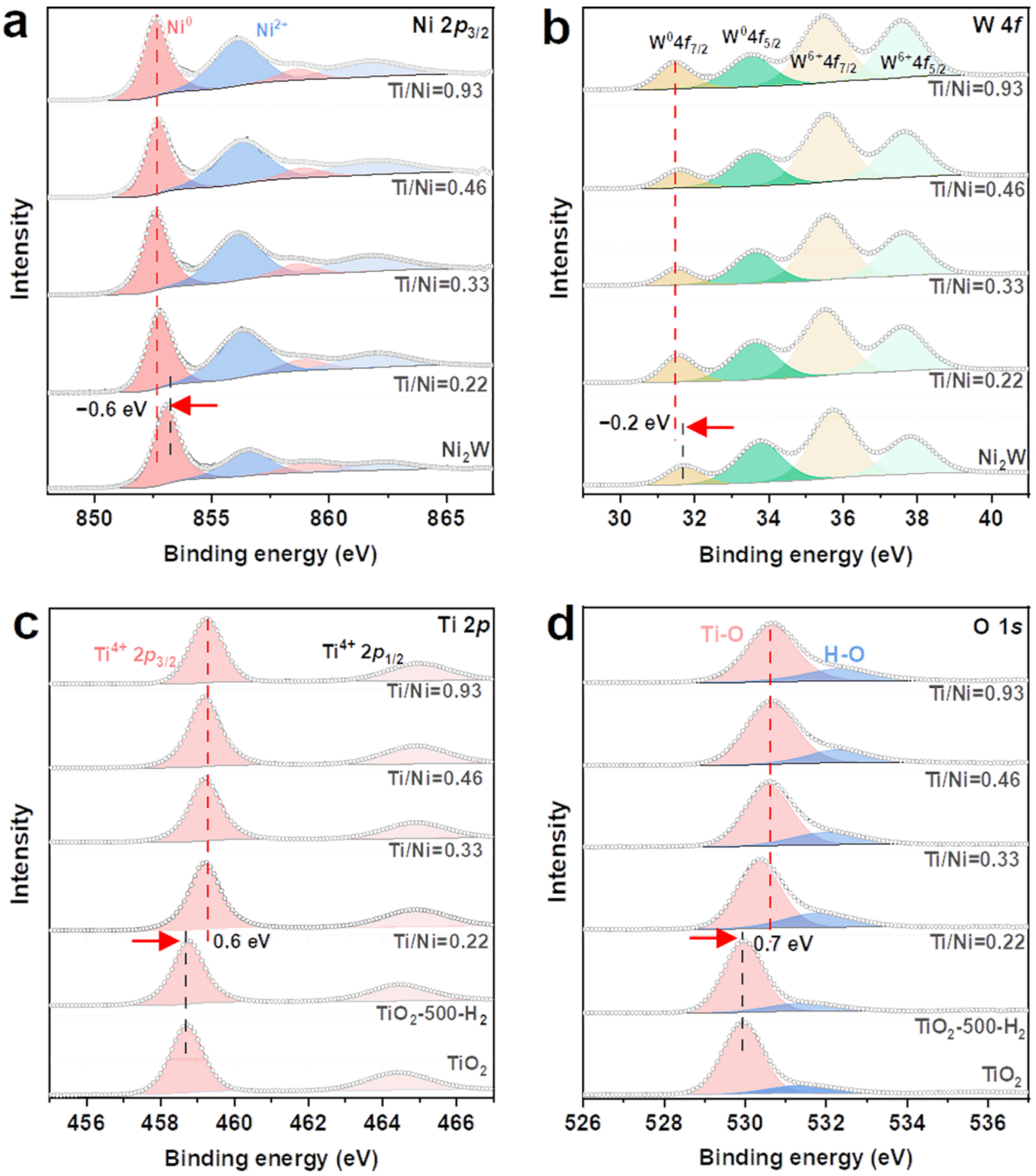

Figure S35. (a) Ni $2p_{3/2}$, (b) W $4f$, (c) Ti $2p$ and (d) O $1s$ level XPS spectra of TiO_2, TiO_2-H_2-500, Ni_2W and Ni_2W/TiO_2 with various Ti/Ni ratios.

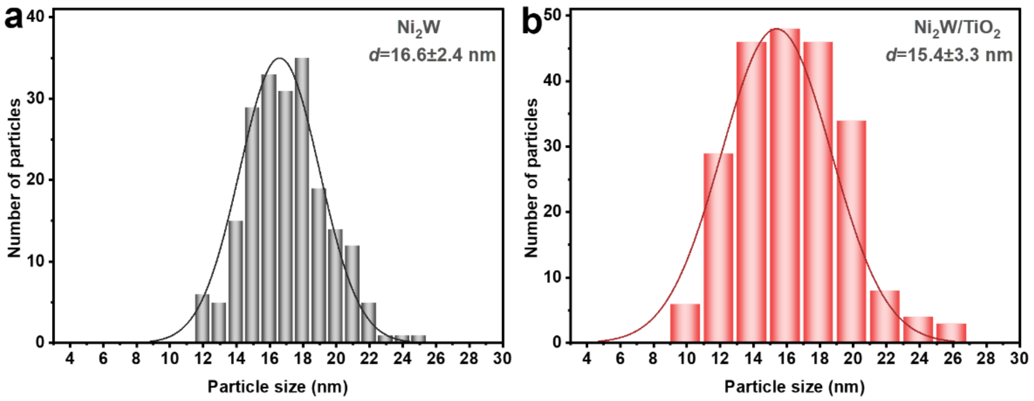

Figure S36. The Ni_2W particle size distributions of (a) Ni_2W and (b) Ni_2W/TiO_2 catalysts. The error bars are standard deviations of the particle size based on at least 200 counts.

.

Table S7. Ni/W molar ratios and W dissolution depths of Ni_2W and Ni_2W/TiO_2 before and after the CV and CA tests.

Sample	Ni/W			W dissolution depth estimated from the ICP-OES result (nm)
	XPS	EDS	ICP-OES	
Ni_2W	3.2	2.9	2.9	——
Ni_2W after CV test	5.2	2.3	3.6	1.1
Ni_2W o after CA test	4.6	4.4	3.7	1.2
Ni_2W/TiO_2	2.9	2.0	2.4	——
Ni_2W/TiO_2 after CV test	4.9	2.3	3.7	1.3
Ni_2W/TiO_2 after CA test	3.8	2.4	3.7	1.3

Note: "Ni_2W" and "Ni_2W/TiO_2" samples represent the catalysts before the electrochemical measurements; "Ni_2W after CV test" and "Ni_2W/TiO_2 after CV test" samples represent the catalysts after CV tests in H_2-saturated 0.1 M NaOH from −0.05 to 1.0 V at 0.5 mV s^{-1}; "Ni_2W after CA test" and "Ni_2W/TiO_2 after CA test" samples represent the catalysts after CA tests at 1.2 V for 2 h in H_2-saturated 0.1 M NaOH. The potentials are not *iR*-corrected.

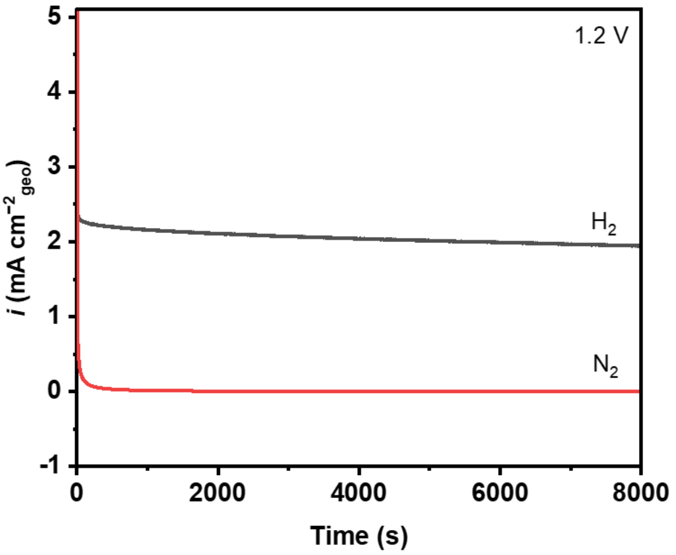

Figure S37. Chronoamperometry curves of Ni_2W/TiO_2 at 1.2 V (no *iR*-correction) in H_2 and N_2-saturated 0.1 M NaOH at 1600 r.p.m. The Ni loading is 312 μg_{Ni} $cm^{-2}{}_{geo}$ for Ni_2W/TiO_2.

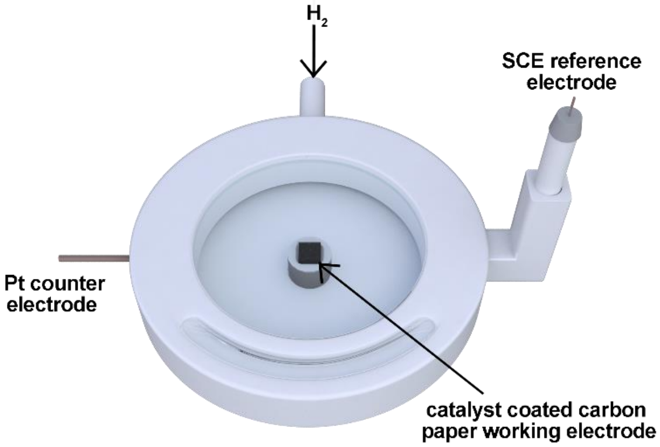

Figure S38. Schematic diagram of the in situ electrochemical Raman cell. The catalyst coated carbon paper, platinum wire and SCE served as the working electrode, counter electrode and reference electrode, respectively. 0.1 M NaOH was used as the electrolyte. H_2 gas was continuously purged into the electrolyte during the test.

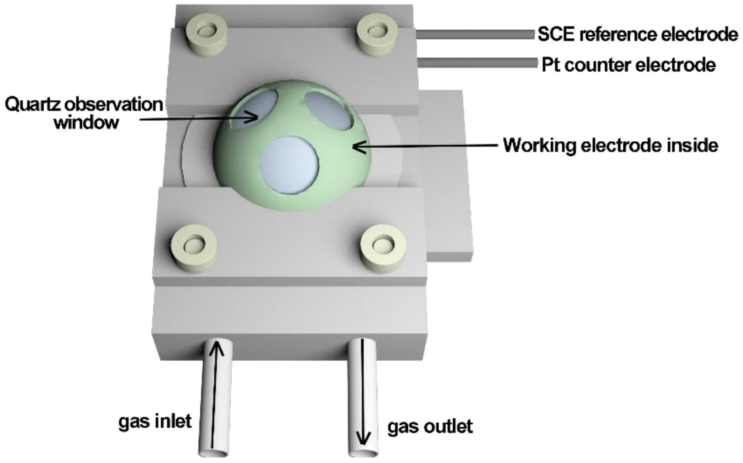

Figure S39. Schematic diagram of the quasi in situ electrochemical XPS cell. The catalyst coated glassy carbon electrode served as the working electrode, which was placed in the center of the cell. The platinum wire and SCE were used as the counter and reference electrodes, respectively. H_2/N_2 mixture saturated 0.1 M NaOH was used as the electrolyte.

Supplementary References

1. Kabir, S. et al. Platinum group metal-free NiMo hydrogen oxidation catalysts: high performance and durability in alkaline exchange membrane fuel cells. *Journal of Materials Chemistry A* **5**, 24433-24443 (2017).
2. Cherstiouk, O. V. et al. Electrocatalysis of the hydrogen oxidation reaction on carbon-supported bimetallic NiCu particles prepared by an improved wet chemical synthesis. *Journal of Electroanalytical Chemistry* **783**, 146-151 (2016).
3. Zhuang, Z. et al. Nickel supported on nitrogen-doped carbon nanotubes as hydrogen oxidation reaction catalyst in alkaline electrolyte. *Nature Communications* **7**, 10141 (2016).
4. Sheng, W. et al. Non-precious metal electrocatalysts with high activity for hydrogen oxidation reaction in alkaline electrolytes. *Energy & Environmental Science* **7**, 1719-1724 (2014).
5. Gao, L. et al. A nickel nanocatalyst within a h-BN shell for enhanced hydrogen oxidation reactions. *Chemical Science* **8**, 5728-5734 (2017).
6. Gao, Y. et al. Improving the antioxidation capability of the Ni catalyst by carbon shell coating for alkaline Hydrogen oxidation reaction. *ACS Applied Materials & Interfaces* **12**, 31575-31581 (2020).
7. Ni, W. et al. Ni_3N as an active hydrogen oxidation reaction catalyst in alkaline medium. *Angewandte Chemie-International Edition* **58**, 7445-7449 (2019).
8. Yang, Y. et al. Enhanced electrocatalytic hydrogen oxidation on Ni/NiO/C derived from a nickel-based metal-organic framework. *Angewandte Chemie-International Edition* **58**, 10644-10649 (2019).
9. Yang, F. et al. Enhanced HOR catalytic activity of PGM-free catalysts in alkaline media: the electronic effect induced by different heteroatom doped carbon supports. *Journal of Materials Chemistry A* **7**, 10936-10941 (2019).
10. Wang, T. et al. Weakening hydrogen adsorption on nickel via interstitial nitrogen doping promotes bifunctional hydrogen electrocatalysis in alkaline solution. *Energy & Environmental Science* **12**, 3522-3529 (2019).
11. Yang, F. et al. Inter-regulated *d*-band centers of the Ni_3B/Ni heterostructure for boosting hydrogen electrooxidation in alkaline media. *Chemical Science* **11**, 12118-12123 (2020).
12. Wang, M. et al. Alloying nickel with molybdenum significantly accelerates alkaline hydrogen electrocatalysis. *Angewandte Chemie-International Edition* **60**, 5771-5777 (2021).
13. Song, F. et al. Interfacing nickel nitride and nickel boosts both electrocatalytic hydrogen evolution and oxidation reactions. *Nature Communications* **9**, 4531 (2018).
14. Duan, Y. et al. Bimetallic nickel-molybdenum/tungsten nanoalloys for high-efficiency hydrogen oxidation catalysis in alkaline electrolytes. *Nature Communications* **11**, 4789 (2020).
15. Deng, S. et al. MoO_2 modulated electrocatalytic properties of Ni: investigate from hydrogen oxidation reaction to hydrogen evolution reaction. *Electrochimica Acta* **324**, 134892 (2019).
16. Yang, F. et al. Boosting hydrogen oxidation activity of Ni in alkaline media through oxygen-vacancy-rich CeO_2/Ni heterostructures. *Angewandte Chemie-International Edition* **58**, 14179-14183 (2019).
17. Song, J. et al. Phase-separated Mo-Ni alloy for hydrogen oxidation and evolution reactions with high activity and enhanced stability. *Advanced Energy Materials* **11**, 2003511 (2021).
18. Deng, S. et al. Insight into the hydrogen oxidation electrocatalytic performance enhancement on Ni via oxophilic regulation of MoO_2. *Journal of Energy Chemistry* **54**, 202-207 (2021).
19. Qin, S. et al. Ternary nickel-tungsten-copper alloy rivals platinum for catalyzing alkaline hydrogen oxidation. *Nature Communications* **12**, 2686 (2021).
20. Su, L. et al. Modification of the intermediate binding energies on Ni/Ni_3N heterostructure for

enhanced alkaline hydrogen oxidation reaction. *Advanced Functional Materials* **31**, 2106156 (2021).
21. Zhao, X. et al. Nitrogen-inserted nickel nanosheets with controlled orbital hybridization and strain fields for boosted hydrogen oxidation in alkaline electrolytes. *Energy & Environmental Science* **15**, 1234-1242 (2022).
22. Gao, Y. et al. A completely precious metal-free alkaline fuel cell with enhanced performance using a carbon-coated nickel anode. *Proceedings of the National Academy of Sciences of the United States of America* **119**, e2119883119 (2022).
23. Men, Y. et al. Oxygen-inserted top-surface layers of Ni for boosting alkaline hydrogen oxidation electrocatalysis. *Journal of the American Chemical Society* **144**, 12661-12672 (2022).
24. Gao, F. Y. et al. Nickel-molybdenum-niobium metallic glass for efficient hydrogen oxidation in hydroxide exchange membrane fuel cells. *Nature Catalysis* **5**, 993-1005 (2022).
25. Ni, W. Y. et al. An efficient nickel hydrogen oxidation catalyst for hydroxide exchange membrane fuel cells. *Nature Materials* **21**, 804-810 (2022).
26. Roy, A. et al. Nickel-copper supported on a carbon black hydrogen oxidation catalyst integrated into an anion-exchange membrane fuel cell. *Sustainable Energy & Fuels* **2**, 2268-2275 (2018).
27. Gu, S. et al. An efficient Ag-ionomer interface for hydroxide exchange membrane fuel cells. *Chemical Communications* **49**, 131-133 (2013).
28. Lu, S. et al. Alkaline polymer electrolyte fuel cells completely free from noble metal catalysts. *Proceedings of the National Academy of Sciences of the United States of America* **105**, 20611-20614 (2008).
29. Hu, Q. et al. Alkaline polymer electrolyte fuel cell with Ni-based anode and Co-based cathode. *International Journal of Hydrogen Energy* **38**, 16264-16268 (2013).
30. Truong, V. M. et al. Platinum and platinum group metal-free catalysts for anion exchange membrane fuel cells. *Energies* **13**, 582 (2020).
31. Du, W. et al. Unveiling the in situ dissolution and polymerization of Mo in Ni_4Mo alloy for promoting the hydrogen evolution reaction. *Angewandte Chemie-International Edition* **60**, 7051-7055 (2021).
32. Tian, H. J., Roberts, C. A. & Wachs, I. E. Molecular structural determination of molybdena in different environments: aqueous solutions, bulk mixed oxides, and supported MoO_3 catalysts. *Journal of Physical Chemistry C* **114**, 14110-14120 (2010).
33. Desilvestro, J., Corrigan, D. A. & Weaver, M. J. Spectroelectrochemistry of thin nickel hydroxide films on gold using surface-enhanced Raman spectroscopy. *Journal of Physical Chemistry* **90**, 6408-6411 (1986).
34. Desilvestro, J., Corrigan, D. A. & Weaver, M. J. Characterization of redox states of nickel hydroxide film electrodes by in situ surface Raman spectroscopy. *Journal of the Electrochemical Society* **135**, 885-892 (1988).
35. Hall, D. S., Bock, C. & MacDougall, B. R. The electrochemistry of metallic nickel: oxides, hydroxides, hydrides and alkaline hydrogen evolution. *Journal of the Electrochemical Society* **160**, F235-F243 (2013).
36. Melendres, C. A. & Pankuch, M. On the composition of the passive film on nickel-a surface-enhanced Raman spectroelectrochemical study. *Journal of Electroanalytical Chemistry* **333**, 103-113 (1992).
37. Hardcastle, F. D. & Wachs, I. E. Determination of molybdenum oxygen bond distances and bond orders by Raman-spectroscopy. *Journal of Raman Spectroscopy* **21**, 683-691 (1990).
38. Durr, R. N. et al. From NiMoO4 to gamma-NiOOH: Detecting the Active Catalyst Phase by Time Resolved in Situ and Operando Raman Spectroscopy. *Acs Nano* **15**, 13504-13515 (2021).

39. Huang, J. et al. Identification of Key Reversible Intermediates in Self-Reconstructed Nickel-Based Hybrid Electrocatalysts for Oxygen Evolution. *Angewandte Chemie-International Edition* **58**, 17458-17464 (2019).
40. Yeo, B. S. & Bell, A. T. In Situ Raman Study of Nickel Oxide and Gold-Supported Nickel Oxide Catalysts for the Electrochemical Evolution of Oxygen. *Journal of Physical Chemistry C* **116**, 8394-8400 (2012).
41. Lo, Y. L. & Hwang, B. J. In situ Raman studies on cathodically deposited nickel hydroxide films and electroless Ni-P electrodes in 1 M KOH solution. *Langmuir* **14**, 944-950 (1998).
